# Mitogenomes of museum specimens provide new insight into species classification and recently reduced diversity of highly endangered *Nomascus* gibbons

**DOI:** 10.1111/1749-4877.12878

**Published:** 2024-07-29

**Authors:** Siqiong LIU, Kexin LI, Yuxin ZHENG, Jiayang XUE, Sheng WANG, Song LI, Peng CAO, Feng LIU, Qingyan DAI, Xiaotian FENG, Ruowei YANG, Wanjing PING, Dongdong WU, Pengfei FAN, Qiaomei FU, Zehui CHEN

**Affiliations:** ^1^ Key Laboratory of Vertebrate Evolution and Human Origins, Institute of Vertebrate Paleontology and Paleoanthropology, Center for Excellence in Life and Paleoenvironment Chinese Academy of Sciences Beijing China; ^2^ College of Earth and Planetary Sciences University of Chinese Academy of Sciences Beijing China; ^3^ College of Life Sciences Northwest University Xi'an Shaanxi China; ^4^ State Key Laboratory of Genetic Resources and Evolution, Kunming Natural History Museum of Zoology Kunming Institute of Zoology, Chinese Academy of Sciences Kunming China; ^5^ Center for Excellence in Animal Evolution and Genetics Chinese Academy of Sciences Kunming China; ^6^ National Resource Center for Non‐Human Primates, Kunming Primate Research Center, and National Research Facility for Phenotypic and Genetic Analysis of Model Animals (Primate Facility) Kunming Institute of Zoology, Chinese Academy of Sciences Kunming China; ^7^ KIZ‐CUHK Joint Laboratory of Bioresources and Molecular Research in Common Diseases Kunming Institute of Zoology, Chinese Academy of Sciences Kunming China; ^8^ School of Life Sciences Sun Yat‐Sen University Guangzhou China

## Abstract

Our findings reveal that the western black crested gibbon (*Nomascus concolor*) did not divide into different subspecies, and the relatively low level of genetic diversity emphasizes the importance of monitoring this indicator for vulnerable wildlife. Meanwhile, phylogeographic analysis of the *Nomascus* genus shows a north‐to‐south trend of ancestral geographic distribution.

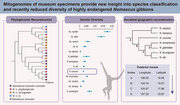

## INTRODUCTION

Gibbons (Hylobatidae) are endangered small arboreal apes distributed in southern, eastern, and Southeast Asia, named for their exceptionally long arms (Fan 2017). Studying gibbons is of paramount importance in evolutionary biology, providing unique insights into the diversification of primates and comprehending hominoid evolution (Carbone *et al.* 2014). According to different karyotypes ranging from 38 to 52, gibbons could be classified into four genera (*Nomascus*, *Hylobates*, *Hoolock*, and *Symphalangus*) with 20 species (Carbone *et al.* 2014; Fan *et al.* 2017). The phylogenetic relationships of the gibbon family remain undetermined, and a variety of different topologies are identified based on various data types or methods. The most frequent topology inferred by mitochondrial sequences is (*Nomascus* (*Symphalangus* (*Hoolock*, *Hylobates*))) (Ortiz *et al.* 2015), while the nuclear genome with more support for *Hoolock* and *Symphalangus* were sister taxa without a resolved unique topology (Carbone *et al.* 2014; Veeramah *et al.* 2015; Shao *et al.* 2023). Among them, the generally accepted first divergent *Nomascus* genus comprises seven distinct species that are divided into two main clades. The first clade includes Hainan gibbon (*N. hainanus*) and eastern black‐crested gibbon (*N. nasutus*), while the other consists of western black‐crested gibbon (*N. concolor*), northern white‐cheeked gibbon (*N. leucogenys*), southern white‐cheeked gibbon (*N. siki*), northern buffed‐cheeked gibbon (*N. annamensis*), and southern yellow‐checked gibbon (*N. gabriellae*) (Thinh *et al.* 2010a, 2010c). All species of *Nomascus* are considered endangered or critically endangered according to *the IUCN Red List* (IUCN 2023). Among these species, *N. hainanus* is considered the rarest primate species in the world, with only about 40 individuals remaining in the Bawangling National Nature Reserve in Hainan, China (Zhong *et al.* 2023), while western black‐crested gibbon (*N. concolor*) recorded 340 groups (∼1500 individuals) found in Yunnan province in China, western Laos, and northern Vietnam (Fang *et al.* 2020; Yang *et al.* 2021; Fan *et al.* 2022). *Nomascus* possess erect crown hairs, of which adult females have very richly colored hairs, while adult males are predominantly black (Mootnick & Fan 2011). Since *Nomascus* fur coloration varies during ontogenesis, taxonomic studies of museum specimens are hindered, whereas obtaining genetic data has been an effective and frequently utilized approach in recent years.

Aside from their survival dilemma, however, the phylogeny and morphology incongruence of subspecies made the classification of *N. concolor* more challenging. Based on their morphological differences (Ma & Wang 1986), *N. concolor* was initially classified into four subspecies: *N. c. concolor*, *N. c. furvogaster*, *N. c. jingdongensis*, and *N. c. lu*. However, the taxonomic status of *N. c. furvogaster* and *N. c. jingdongensis* has been questioned since a phylogenetic analysis based on the dataset of cytochrome b (*cytb*) gene indicates that *N. c. furvogaster* and *N. c. jingdongensis* are reclassified as *N. c. concolor* (Thinh *et al.* 2010b). Although their morphological and song structure differences were not notable, they are designated as independent subspecies in many studies depending on regional field investigations because of the dispersed geographic distribution (Geissmann 2007; Mootnick & Fan 2011; Fan 2017). These lingering uncertainties sparked public interest, but they remain unresolved due to a paucity of available samples. In this case, museum specimens provide a window to studying the gibbon evolutionary episodes, particularly species history sitting in deep ancestral branches of the phylogeny (Thinh *et al.* 2010b).

## MATERIALS AND METHODS

### DNA extraction and sequencing

We obtained nine museum skin specimens (from AD 1957 to AD 1983) including eight *N. concolor* and one *N. hainanus* from Kunming Institute of Zoology and Museum of Sun Yat‐sen University, respectively (for details, see Table S1, Supporting Information). DNA extraction of the gibbon museum samples was carried out in the Laboratory of Molecular Paleontology at the Institute of Vertebrate Paleontology and Paleoanthropology, Chinese Academy of Sciences, and followed strict ancient DNA standards and cleaning procedures. Negative controls were added for DNA extraction and all polymerase chain reactions. The working surfaces and experiments were purified with bleach and/or ultraviolet irradiation to avoid bench contamination. Museum skin specimens were cut into <1 mm^3^ pieces, rinsed with 70% ethanol, and used for DNA extraction following the protocol described by Dabney *et al.* (Dabney *et al.* 2013; Wang *et al.* 2019; Zhang *et al.* 2021). We constructed double‐stranded libraries and treated them with half or full uracil‐DNA‐glycosylase treatment to remove damaged bases. Thirty cycles of DNA amplification were performed in the presence of Pfu Turbo Cx Hotstart DNA Polymerase. To identify present‐day human contamination, sample‐specific indexes were introduced into P5 and P7 adapters during the library amplification step (Kircher *et al.* 2012). DNA concentration of each sample was assayed with a NanoDrop 2000 spectrometer. The libraries were subjected to high‐throughput sequencing using 2 × 76‐bp reads on the Illumina Miseq platform or using 2 × 150‐bp reads on the Illumina HiSeq X platform, followed by merging of overlapping mate‐pairs and trimming of the adapter using leeHom (https://github.com/grenaud/leeHom; Renaud *et al.* 2014) with the parameter “‐ancientdna.” Finally, we used BWA v0.5.10 (Li & Durbin 2009) to map clean reads to the gibbon reference mitogenome (Genbank accession number: NC_021 957) and then removed mapped fragments smaller than 30 bp and removed duplicates using bam‐rmdup (version: 0.6.3, https://bitbucket.org/ustenzel/biohazard‐tools). To estimate the contamination rate of the samples, we used 311 present‐day human mitochondrial genomes as potential contaminants and compared them to the sequencing data using contamMix (Fu *et al.* 2013).

### Dataset construction

We built a full‐length mtDNA and *cytb* dataset to reconstruct the phylogeny of the gibbon family. In total, we collected 100 complete mitochondrial genomes downloaded from the National Center for Biotechnology Information (NCBI), of which 83 were from modern gibbons from all four genera (*Nomascus*, *Hylobates*, *Hoolock*, and *Symphalangus*), including 14 species (for detailed information, see Table S2, Supporting Information). We took 17 mitochondrial genomes from other primates (Eastern gorilla, *Gorilla beringei*; western gorilla, *Gorilla gorilla;* human, *Homo sapiens*; chimpanzee, *Pan troglodytes*; bonobo, *Pan paniscus*; Sumatran orangutan, *Pongo abelii*; Bornean orangutan, *Pongo pygmaeus*; olive baboon, *Papio anubis*; and rhesus macaque, *Macaca mulatta*) as outgroups. Together with our new historical samples, there are 109 complete mitochondrial genomes used in subsequent analyses. We used MAFFT v7.490 to generate multiple genome alignments (Katoh & Standley 2013). To increase the accuracy of phylogenetic analysis, we filtered the badly mapped sites (i.e. gap site including “N” and “‐”) by setting 10%, 30%, and 50% as the filter thresholds, respectively (x% means that if more than x% of the sequences at this site are gap or ambiguous base, the site will be discarded). Finally, we only kept the phylogenetic results generated from the alignment that filtered 30% badly mapped sites (with 16 437 sites remained), as it kept the most of effective polymorphism sites and the tree topology become more stable. Because the available full‐length mtDNA dataset does not contain all species of the gibbon family, we also collected a total of 400 *cytb* sequences covering all the species of the gibbon family, consisting of 107 split *cytb* segments from full‐length mtDNA sequences and 293 added *cytb* sequences of gibbon downloaded from NCBI (for corresponding accession number, see Table S2, Supporting Information).

### Investigation of phylogenetic relationships among gibbons

Bayesian phylogenetic trees were constructed by BEAST v1.10.4 (Suchard *et al.* 2018). We used jModeltest v2.1.10 (Posada 2008) to test the best‐fit nucleotide substitution model for full‐length and *cytb* sequences. The best‐fit models were both GTR + I + G under a Bayesian information criterion. To choose a suitable molecular clock, we first tried an uncorrelated lognormal relaxed clock and found that the posterior density of lognormal standard deviations was close to zero, so we chose a strict molecular clock. We then tested the best tree prior by estimating log marginal likelihoods using the path‐sampling (PS) and stepping‐stone sampling (SS) methods (Baele *et al.* 2012). The results support that the best tree prior among the three tested models (i.e. constant size, Skyline piecewise‐constant, and Skyline piecewise‐linear) is Skyline piecewise‐constant (PS and SS values: −128974.7 and −128982.9), followed by the Skyline piecewise‐linear (PS and SS values: −128984.2 and −128986.7) and constant size (PS and SS values: −129003.5 and −129003.3) (Ko *et al.* 2018). We used three fossil calibration points of the order Primates: (1) Time to the most recent common ancestor (TMRCA) of genus *Homo‐Pan* (mean = 6.5 Mya, SD = 0.8 Mya); (2) TMRCA of subfamily *Homininae‐Ponginae* (mean = 15.5 Mya, SD = 2.5 Mya); (3) TMRCA of parvorder *Catarrhini* (mean = 29.0 Mya, SD = 6.0 Mya) (Zhang *et al.* 2019). We ran the 50 million Markov chain Monte Carlo (MCMC) chains, sampling every 5000 steps, to ensure that the effective sample size values were greater than 300. We discarded 10% of the trees as burn‐in using TreeAnnotator v1.10.4 (Suchard *et al.* 2018; Zhu *et al.* 2022). The final tree was visualized in FigTree v1.4.4 (http://tree.bio.ed.ac.uk/software/figtree/). In addition, to verify the accuracy of tree topologies, we utilized complete genomes and different mtDNA partitions to reconstruct phylogenetic trees and estimate the divergence time (for details, see Table S3, Supporting Information). We extracted rRNAs (2508 sites), 13 protein‐coding genes (11 406 sites; the stop codons were completed according to the reference sequence annotation, and ND6 were reversed complementary), first and second codon positions of protein‐coding genes (7604 sites), and third codon positions of protein‐coding genes (3802 sites). According to the jModeltest v2.1.10 test, the best‐fit nucleotide substitution model for these sequences was still GTR + I + G. For the full‐length mtDNA and the *cytb* dataset, we also constructed a maximum likelihood (ML) tree using RAxML v8.1.12 (Stamatakis 2014), with GTRGAMMI as the nucleotide substitution model and bootstrapped 1000 replicates.

### Haplotype network construction, 3D PCA, and unique mutation analysis

To further analyze the genetic relationships among four subspecies of *N. concolor*, we used DnaSP v6.12.03 (Rozas *et al.* 2017) to determine the haplotypes for full‐length mtDNA and the *cytb* gene (Zhang *et al.* 2020). Ninety‐one sites from the full‐length mtDNA were detected and clustered into 8 haplotypes, while 26 sites from *cytb* were classified into 19 haplotypes. Then, we visualized the results over the median‐joining method by Popart v1.7 (Bandelt *et al.* 1999). Single nucleotide polymorphisms (SNPs) of the individuals were called using the fasta2genlight function of the adegenet package in R (Jombart & Ahmed 2011; Zhang *et al.* 2022). Subsequently, the SNPs were downscaled using principal component analysis (PCA), and 3D scatterplots were generated using the scatterplot3d package in R (Ligges & Maechler 2003). We divided the sequences from the *Nomascus* genus into two groups, *N. concolor* and all sequences except *N. concolor*, and then obtained the base concordant sites of each group. After discarding the same sites of the two groups' base concordant, we finally identified the unique mutation in *N. concolor*. The same method was used for other species except *N. hainanus*, which was sequenced at a lower depth.

### Pairwise genetic distance and genetic diversity estimation

Pairwise genetic distances between individuals within *N. concolor* were estimated using MEGA v11.0.13 (Tamura *et al.* 2021) with default parameters by taking 1000 bootstraps. The p‐distance matrix was processed into a heatmap using the pheatmap package in R (Kolde 2019, https://CRAN.R‐project.org/package=pheatmap). Segregation sites (*S*), haplotype number (*H*), haplotype diversity (*H*_d_), nucleotide diversity (π), and average number of nucleotide differences (*k*) were calculated using DnaSP v6.12.03. Two theta estimates ($\theta_{S}$, from the observed number of segregating sites;$\;\theta_{\pi }$, from the mean number of pairwise differences in nucleotide diversity) were calculated using Arlequin v3.5.2 (Excoffier *et al.* 2005), and the female effective population size (*N*_ef_) was calculated with reference to Salado *et al.* (2022). Generation time (*g* = 15) was referenced by the International Union for Conservation of Nature (IUCN 2023).

### Selection pressure estimation using protein‐coding genes

To test whether *N. concolor* and *N. hainanus* are under different selection pressures compared to other gibbons within the *Nomascus* genus, we calculated ω (i.e. *d*N/*d*S, nonsynonymous/synonymous ratio) for concatenated 13 protein‐coding genes using the CODEML program in PAML 4.9 (Yang 2007). We used the simplest model M0, which assumes that all branches and sites with one ω, as the null hypothesis. Alternative hypotheses used *N. concolor* and *N. hainanus* as foreground branches, respectively. A chi‐squared test then was performed using twice the log‐likelihood difference between the null and alternative hypotheses. In addition, for the internal branching of *N. concolor*, we tested three alternative hypotheses using the Branch model based on different groupings, for example, grouping with geographic locations, subspecies classification, and phylogenetic branches.

### Ancestral geographic reconstruction within *Nomascus* genus

Reconstruction of the geographic distribution of ancestral nodes within *Nomascus* was estimated using the geo model in BayesTraits v4.0.0 (http://www.evolution.reading.ac.uk/BayesTraitsV4.0.0/BayesTraitsV4.0.0.html) based on the *cytb* dataset. The geo model is based on Brownian motion by mapping longitude and latitude onto a three‐dimensional Cartesian coordinates system. To run BayesTraits v4.0.0, a phylogenetic tree with time and the corresponding geo‐information of each sample is required. Therefore, we collected latitude and longitude information for each individual and reconstruted a phylogenetic tree using sequences for which geographic information was available using BEAST v1.10.4 as the input file. The geo model produces 1000 pairs of ancestral geographic coordinates after running an MCMC of 1 000 000 iterations. The geographic coordinates were mapped to maps for kernel density analysis in ArcGIS 10.6. To investigate whether any climatic or geographic factors affected the past distribution of gibbons, we first collected the spatiotemporal information of gibbon fossils that could be assigned to the extant genera (see Table S4, Supporting Information). In addition, the historical distribution of gibbons was retrieved from Zhou and Zhang (2013), who summarized the records of gibbon occurrence in China over the past 500 years based on ancient literature. Data of five climatic or geographic variables (i.e. mean temperature, precipitation, relative humidity, sunshine, and elevation) are collected from the CRU CL (version 2.0) of the Climate Research Units, Norwich, a data set of station means for the period centered on 1961–1990 (New *et al.* 2002). We calculated the mean values of these factors for each station, performed the ordinary Kriging interpolation analysis, and then projected the occurrence sites onto the geospatial map based on different climatic or geographic factors using ArcGIS Pro 3.2.0. The corresponding values of each factor for the occurrence records of gibbons were extracted and plotted with the ggplot2 package (Wickham 2016) in R version 4.3.2 (R Core Team 2023). We also conducted a significance test using the Mann–Whitney U test to investigate whether there is a difference between the distribution of these values in the Holocene and the historical period (Mann & Whitney 1947).

## RESULTS AND DISCUSSION

Using a modified ancient DNA extraction technique upgraded for museum samples (Zhang *et al.* 2021), in total, we generated eight high‐coverage complete mitochondrial genomes for *N. concolor*, with coverage varying from 85.68 to 1659.93 folds, and one for *N. hainanus* with 1.37 folds, which is the first mitogenome of the species. All mitochondrial genome sequences reported in this paper have been deposited in the GenBase in the National Genomics Data Center (National Genomics Data Center Members and Partners (CNCB‐NGDC) 2023), Beijing Institute of Genomics, Chinese Academy of Sciences/China National Center for Bioinformation, Bioproject number PRJCA023216 that is publicly accessible at https://ngdc.cncb.ac.cn/genbase.

### Mitochondrial evidence supports the non‐subspecific divergence of the *N. concolor*

**Figure 1:**
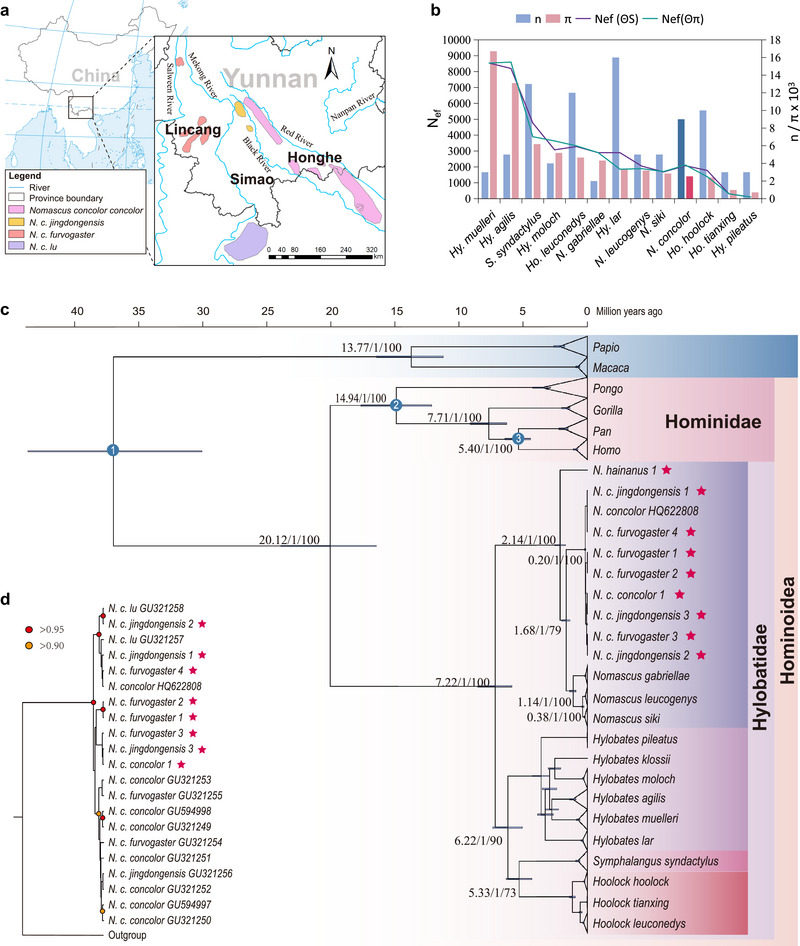
(a) Distribution of *Nomascus concolor* and the location of sampling sites. For a more explicit description, the classification of four distinct subspecies of *N. concolor* was followed in this study. (b) Nucleotide diversity (π), number of sequences (*n*), and the female effective population size (*N*_ef_) using full‐length mtDNA sequences. *N. concolor* is highlighted in the deepened colors. (c) The phylogenetic tree constructed by BEAST and RAxML of gibbons based on full‐length mitochondrial DNA taking *Gorilla*, *Homo*, *Pan*, *Pongo*, *Papio*, and *Macaca* as the outgroups. Our samples are marked by pentagrams. The numbers near the nodes represent divergence time, posterior probability of Bayesian inference, and bootstrap value measured by maximum likelihood methods, respectively. The blue circles denote the three fossil calibration points. (d) The Bayesian tree reconstructed from the *cytb* gene within the *Nomascus* genus, and for the Bayesian tree built from the *cytb* gene using the whole dataset, which including sequences from the gibbon family, see Fig. S3, Supporting Information.

To resolve the puzzles of phylogenetic incongruence among subspecies of *N. concolor*, multiple methods were applied for phylogenetic reconstruction using mitochondrial full‐length and different partition sequences (Fig. 1c,d; Figs S1–S3, S8–S11, Supporting Information). Unlike previous investigations, our findings indicated that *N. concolor* was a single clade, but the phylogenetic relationships of other *Nomascus* species are consistent with previous research (Chan *et al.* 2010; Thinh *et al.* 2010a, 2010b), and all nine individuals generated in this study clustered with corresponding published *N. concolor* or *N. hainanus* samples. *N. concolor* did not form any separate clades according to the subspecies named and characterized in the previous studies (Ma & Wang 1986). *N. c. lu* was previously considered as a determined subspecies (Thinh *et al.* 2010b); two individuals of this subspecies, however, clustered with *N. c. jingdongensis* and *N. c. furvogaster*, which was consistent with the results of pairwise genetic distance analysis (Fig. 1d; Fig. S4, Supporting Information). To validate those findings, we further applied haplotype network reconstruction using the *cytb* gene, which showed that the four previously defined subspecies clustered into a single clade as well and held minimum different sites between each other (Fig. S5, Supporting Information). We also performed PCA based on SNP data, which supported that the four subspecies were not significantly distinct from a maternal perspective (Fig. S6, Supporting Information).

### Estimation of divergence times

We computed the mean substitution rate at 1.333 × 10^−8^ (95% HPD, 1.10 × 10^−8^–1.59 × 10^−8^) substitutions/site/year based on the dataset of complete mitogenomes. With this substitution rate, the divergence time between gibbons and other apes was estimated as 20.12 Mya, which is similar to the previous studies (16.26–21.73 Mya) (Thinh *et al.* 2010a; Fan *et al.* 2017; Shao *et al.* 2023). Within the four genera of gibbon, *Nomascus* genus first diverged from the Hylobatidae family around 7.22 Mya (95% HPD, 5.90–8.59 Mya), while the latest divergence occurred between *Hoolock* and *Symphalangus* at 5.33 Mya (95% HPD, 4.34–6.37 Mya), in line with the Late Miocene global cooling event (Wen *et al.* 2023). The divergence time of *N. hainanus* and the lineage in which *N. concolor* was located was ∼2.14 Mya (95% HPD, 1.72–2.57 Mya) and *N. concolor* diverged from *N. leucogenys*, *N. siki*, and *N. gabriellae* at ∼1.68 Mya (95% HPD, 1.36–2.01 Mya; Fig. 1 and Table S3, Supporting Information). We found that the TMRCA of *N. concolor* lineage can be traced back to 0.20 Mya (95% HPD, 0.15–0.25 Mya), coincident with when the Penultimate Glaciation (0.13–0.30 Mya) emerged in China (Zheng *et al.* 2002). The estimated divergence times based on different partitions (e.g. *cytb* genes, concatenated 13 protein‐coding genes, 1st–2nd codon positions, 3rd codon position, and rRNAs) are similar to those obtained from the full‐length mtDNA (for detailed information, please see Table S3, Supporting Information).

### Genetic diversity loss of *N. concolor* and selection pressure on *Nomascus* genus

Genetic diversity is an indicator of the ability of a species or population to adapt to a changing environment (Hu 2015; Grenier *et al.* 2016). Even if a species has moderate to high population size, if the genetic diversity is low, it is vulnerable and needs to be protected. Therefore, evaluating the genetic diversity of threatened animals is a crucial undertaking. To comprehensively assess the level of genetic diversity within gibbons, we estimated multiple diversity indexes using whole mitochondrial genomes of each species. Overall, among all the gibbons, *Hylobates* have the widest diversity range from 0.00071 to 0.01671, while *Nomascus* is narrowest from 0.00432 to 0.00253, among which *N. concolor* exhibited a relatively low level of nucleotide diversity (π = 0.00253) within gibbons, which is consistent with a previous study about three *N. concolor* populations that inhabit the Wuliang Mountains (with a nucleotide diversity of 0.00364 for the D‐loop region) (Fig. 1b; Table S5, Supporting Information) (Hu 2015). Compared to other Critically Endangered primates, the genetic diversity of *N. concolor* estimated by D‐loop sequences was comparable to that of the gray snub‐nosed monkey (*Rhinopithecus brelichi*; π = 0.00413) and significantly lower than that of the white‐headed langur (*Trachypithecus leucocephalus*; π = 0.01167) (Table S6, Supporting Information). The low level of genetic diversity maintained in the gibbon family may be caused by the long‐term effect of the rapid decline of primates’ effective population size (*N_e_*) during the Late Pleistocene (Shao *et al.* 2023) and has been exacerbated by the contraction and fragmentation of their habitats in recent decades (Cheng 2017).

To further testify to the intraspecific diversity of *N. concolor*, we divided *N. concolor* samples into five groups according to their geographical locations. Surprisingly, we found that the Simao group had the highest nucleotide diversity as measured by the *cytb* gene (π = 0.00661 ± 0.00198) (Table S7, Supporting Information); nevertheless, the last observations of *N. concolor* in Simao were recorded before 1986 (Ni & Ma 2006). This suggested that at least one diversified population might once have lived in Simao and had long since disappeared. Furthermore, 176 unique mutation sites were detected in *N. concolor*, while we detected 121 (*N. gabriella*e), 14 (*N. leuconedys*), and 19 (*N. siki*) unique mutation sites in the other 3 species of *Nomascus*, respectively (Fig. S7, Supporting Information). To measure whether *N. concolor* and *N. hainanus* were under different selection pressures, a *d*N/*d*S ratio analysis was carried out. The results of the log‐likelihood test indicated that the null hypothesis fits the data better than the alternative hypotheses tested with *N. concolor* and *N. hainanus* as foreground branches, respectively, suggesting that these two species were not under different selection pressures (Table S8, Supporting Information). Similarly, all branches of *N. concolor* are under similar purifying selection (ω = 0.09754), as alternative hypotheses could not reject the null hypothesis (Table S9, Supporting Information).

### Ancestral geographic status estimation of *Nomascus*

To date, *Nomascus* is restricted to its native habitats in southern China (especially Yunnan), eastern Cambodia, Vietnam, and Laos (Thinh *et al.* 2010b). The most recent records of *N. concolor* were discovered in the Biluo Snow Mountains, representing the most northerly known population (Fang *et al.* 2020). Early fossil records of *Nomascus* were found in Guangxi, such as Baikong Cave and Sanhe Cave, which could be dated back to the early Pleistocene (Table S4, Supporting Information) (Zhang *et al.* 2018). Combined with the results of the phylogenetic tree, it appears that *Nomascus* may have originated in a relatively northern region and then dispersed southward (Thinh *et al.* 2010c). To investigate the migration patterns of this genus, the modeling of the geographical distribution for ancestral nodes was carried out. We found that along with the selected ancestral nodes within the *Nomascus* genus becoming younger, the posterior mean values of their latitude decreased, suggesting a southward dispersal (Fig. S12, Supporting Information). Furthermore, to find the drivers of the potential gibbon migration, we gathered gibbon fossils and historical occurrence sites in China (Table S4, Supporting Information). The results show that gibbon distribution has not significantly changed on large time scales, which we grouped into four intervals, that is, Pliocene and Pleistocene, Late Pleistocene, Holocene, and Historical. Projection of fossils or historical records onto the distribution bands of five climatic or geographic factors (including mean temperature, precipitation, relative humidity, sunshine, and elevation) shows that all the climatic or geographic factors do not make a significant impact on the distribution of gibbons in different time periods, suggesting that gibbons are well adapted to their environment (Figs S13–S18, Supporting Information). Thus, the recent decline in gibbon distribution might be primarily due to human activities but not climate change since the Holocene climate became stable (Zhao *et al.* 2023).

## CONCLUSION

In conclusion, our sampling, sequencing, and analysis of nine gibbon mitogenomes have provided numerous insights into enhancing the resolution of species classification via museum samples and increased the knowledge of the genetic diversity patterns of gibbons. First, thanks to the newly adopted samples and sequenced full mitogenomes within the *Nomascus* genus, we clarified the mitogenomic phylogeny discordancy at a subspecies level and, for the first time, estimated the divergence time of *N. concolor*. Second, the lasting low genetic diversity within the *Nomascus* genus enlightens us that, for threatened animals, there is a great necessity to increase the focus on genetic diversity in addition to monitoring their population size. Third, we observed a southward dispersal trend of *Nomascus*, which is consistent with the previous speculation based on the divergence order of the North and South lineages within *Nomascus* species. As our study was limited by the available data and the sampling range, further extensive sampling of *N. concolor* would be required for deeper exploration. Together, these results advanced our knowledge of the matrilineal genetic characteristics of the *Nomascus* genus and highlighted the complex speciation episodes of these species, providing insights into the species classification and the dynamic changes of their ancestral states.

## Supporting information


**Figure S1** Maximum likelihood tree constructed from the whole mitochondrial genomes.
**Figure S2** Maximum likelihood tree constructed from the *cytb* gene.
**Figure S3** Bayesian phylogenetic tree constructed from the *cytb* gene.
**Figure S4** Pairwise genetic distances between individuals within *N. concolor*.
**Figure S5** Haplotype network of the *cytb* gene in *N. concolor*.
**Figure S6** Principal component analysis (PCA) based on SNPs of *cytb* gene.
**Figure S7** Number of unique mutation sites of four *Nomascus* species.
**Figure S8** Bayesian phylogenetic tree based on the protein‐coding sequences (CDS).
**Figure S9** Bayesian phylogenetic tree based on rRNAs.
**Figure S10** Bayesian phylogenetic tree based on 1^st^‐2^nd^ codon.
**Figure S11** Bayesian phylogenetic tree based on 3^rd^ codon.
**Figure S12** Putative ancestral distribution of phylogenetic nodes within *Nomascus*.
**Figure S13** Fossil sites and historical distribution of gibbons in China relative to temperature.
**Figure S14** Fossil sites and historical distribution of gibbons in China relative to precipitation.
**Figure S15** Fossil sites and historical distribution of gibbons in China relative to humidity.
**Figure S16** Fossil sites and historical distribution of gibbons in China relative to sunshine.
**Figure S17** Fossil sites and historical distribution of gibbons in China relative to elevation.
**Figure S18** The distribution of climatic or geographic factor values associated with Holocene and historical records of gibbons.

**Table S1** New samples sequenced for this study
**Table S2** Mitochondrial full‐length or *cytb* sequences from published papers
**Table S3** Comparison of estimated divergence times for different partitions
**Table S4** Fossil sites of gibbon family used in our study
**Table S5** Diversity indices calculated using mitochondrial genomes for different species in gibbon family
**Table S6** Comparison of D‐loop genetic diversity with other primates
**Table S7** Diversity indices for different sites calculated by *cytb*
**Table S8** Selection pressures on *N. concolor* and *N. hainanus*
**Table S9** Selection pressures on different branches of *N. concolor*
