# Supplementary material for: Mitogenomes of museum specimens provide new insight into species classification and recently reduced diversity of highly endangered Nomascus gibbons
Source: Integr Zool. 2024 Jul 29;20(3):674–84. doi: 10.1111/1749-4877.12878 (PMC12046444; doi:10.1111/1749-4877.12878)
Supplement: Supplementary file 1 — Figure S1 Maximum likelihood tree constructed from the whole mitochondrial genomes. Figure S2 Maximum likelihood tree constructed from the cytb gene. Figure S3 Bayesian phylogenetic tree constructed from the cytb gene. Figure S4 Pairwise genetic distances between individuals within N. concolor. Figure S5 Haplotype network of the cytb gene in N. concolor. Figure S6 Principal component analysis (PCA) based on SNPs of cytb gene. Figure S7 Number of unique mutation sites of four Nomascus species. Figure S8 Bayesian phylogenetic tree based on the protein‐coding sequences (CDS). Figure S9 Bayesian phylogenetic tree based on rRNAs. Figure S10 Bayesian phylogenetic tree based on 1st‐2nd codon. Figure S11 Bayesian phylogenetic tree based on 3rd codon. Figure S12 Putative ancestral distribution of phylogenetic nodes within Nomascus. Figure S13 Fossil sites and historical distribution of gibbons in China relative to temperature. Figure S14 Fossil sites and historical distribution of gibbons in China relative to precipitation. Figure S15 Fossil sites and historical distribution of gibbons in China relative to humidity. Figure S16 Fossil sites and historical distribution of gibbons in China relative to sunshine. Figure S17 Fossil sites and historical distribution of gibbons in China relative to elevation. Figure S18 The distribution of climatic or geographic factor values associated with Holocene and historical records of gibbons. [file INZ2-20-674-s002.docx]

SUPPLEMENTARY MATERIALS

Supplementary Figures


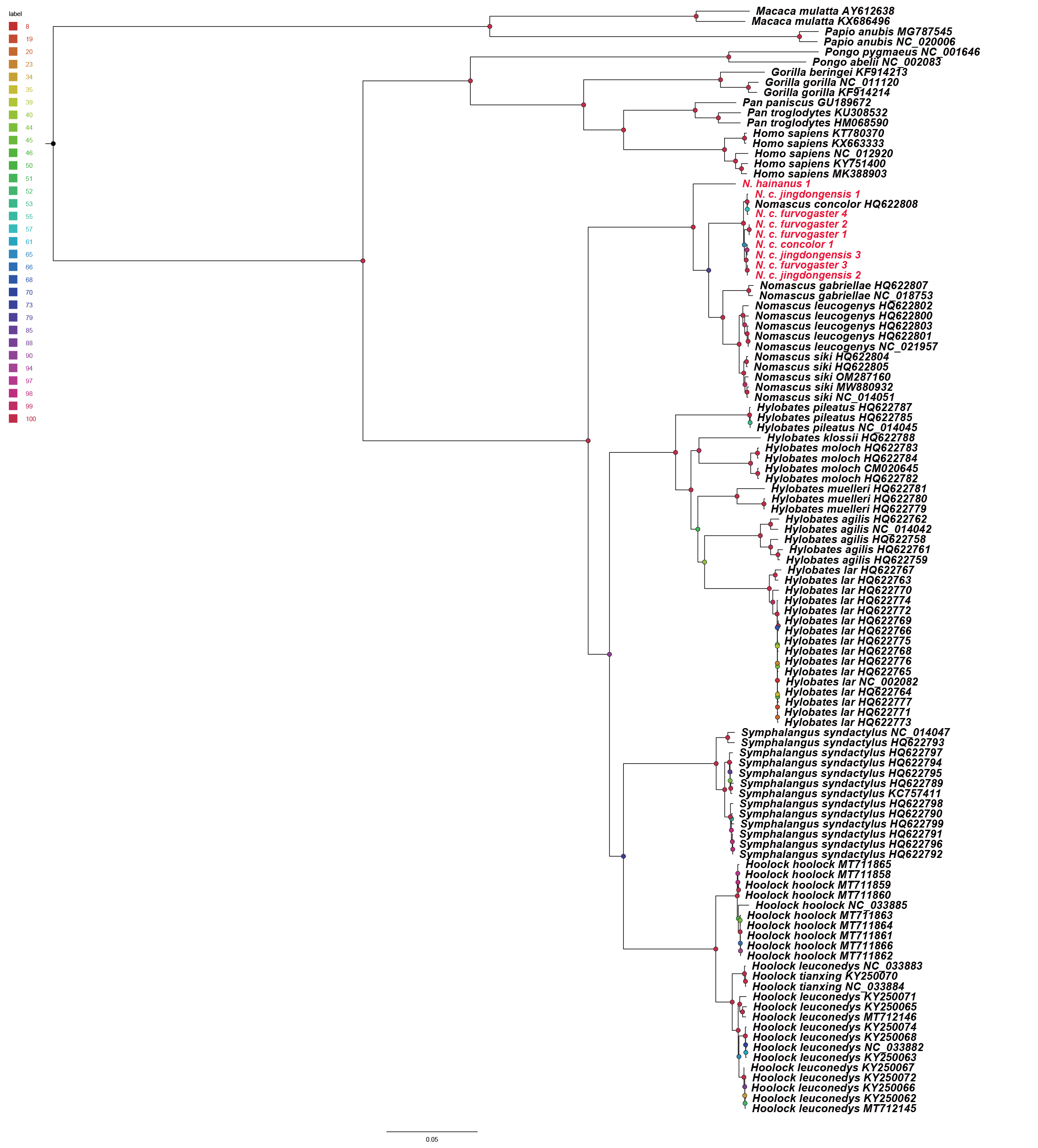


Figure S1 Maximum likelihood tree constructed from the whole mitochondrial genomes. New produced samples in this study are shown in red. Colored dots on nodes indicate the bootstrap values.


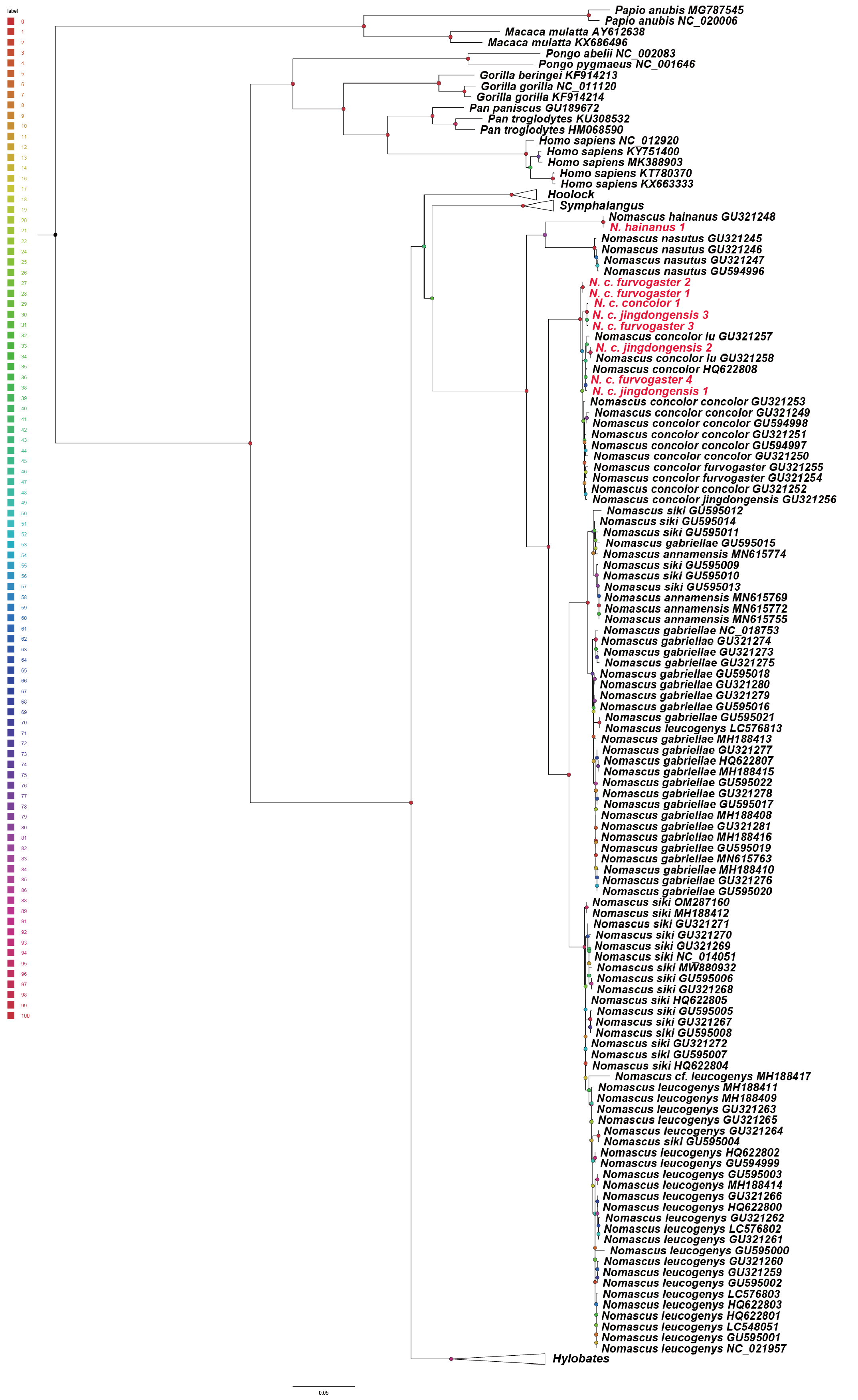


Figure S2 Maximum likelihood tree constructed from the *cytb* gene. New produced samples in this study are shown in red. Colored dots on nodes indicate the bootstrap values. Here, genus of *Hoolock*, *Symphalangus*, and *Hylobates* were merged into a single clade.


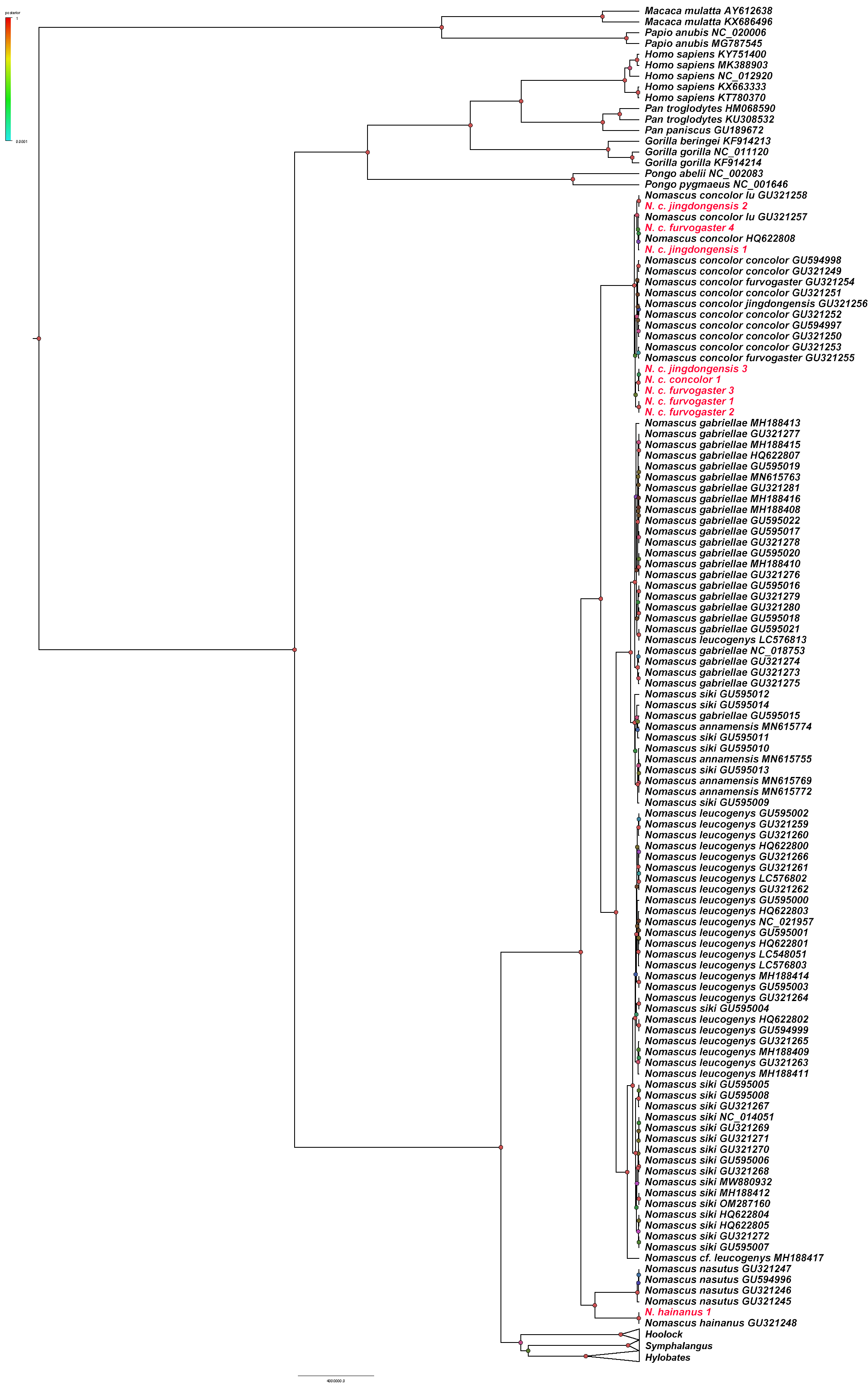


Figure S3 Bayesian phylogenetic tree constructed from the *cytb* gene. New produced samples in this study are shown in red. Colored dots on nodes indicate the posterior probability. Here, genus of *Hoolock*, *Symphalangus*, and *Hylobates* were merged into a single clade.


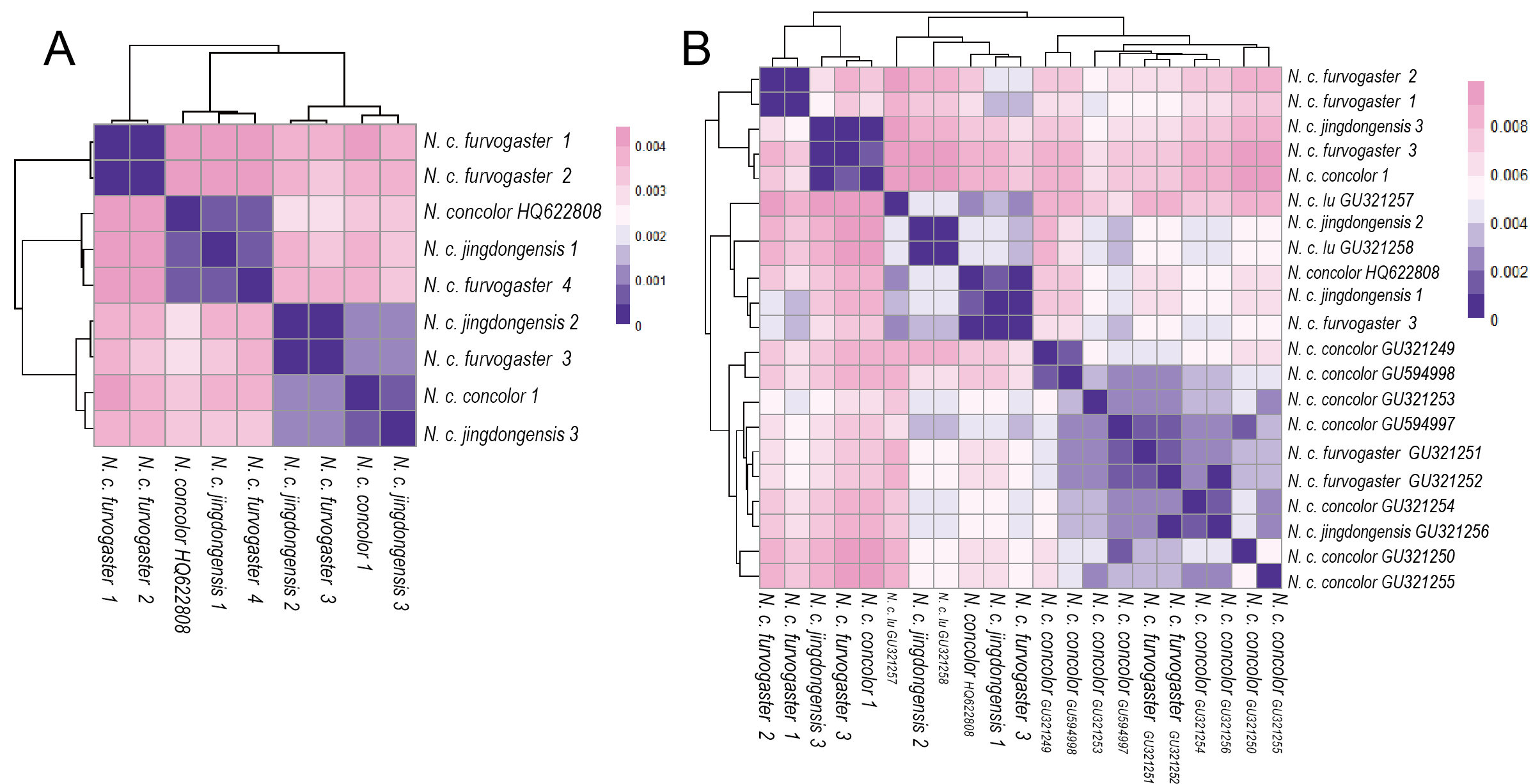


Figure S4 Pairwise genetic distances between individuals within *N. concolor*. Estimation based on (A) complete mitochondrial genomes and (B) *cytb* sequences, respectively.


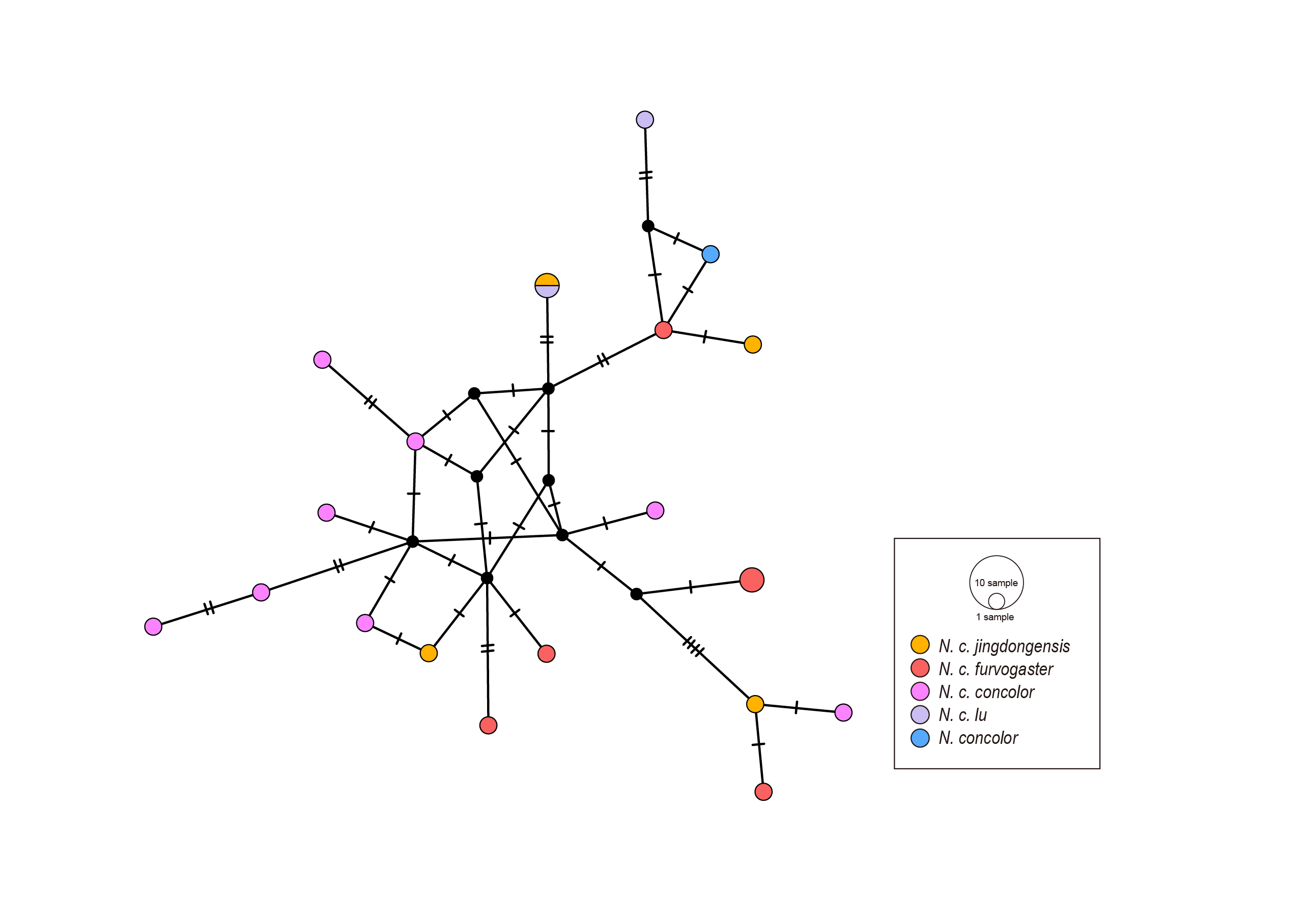


Figure S5 Haplotype network of the *cytb* gene in *N. concolor*. The haplotype network was constructed using median-joining method. The color of circles depends on subspecies and its size correlates with the number of individuals. Short lines on the connecting lines represent mutations between haplotypes.


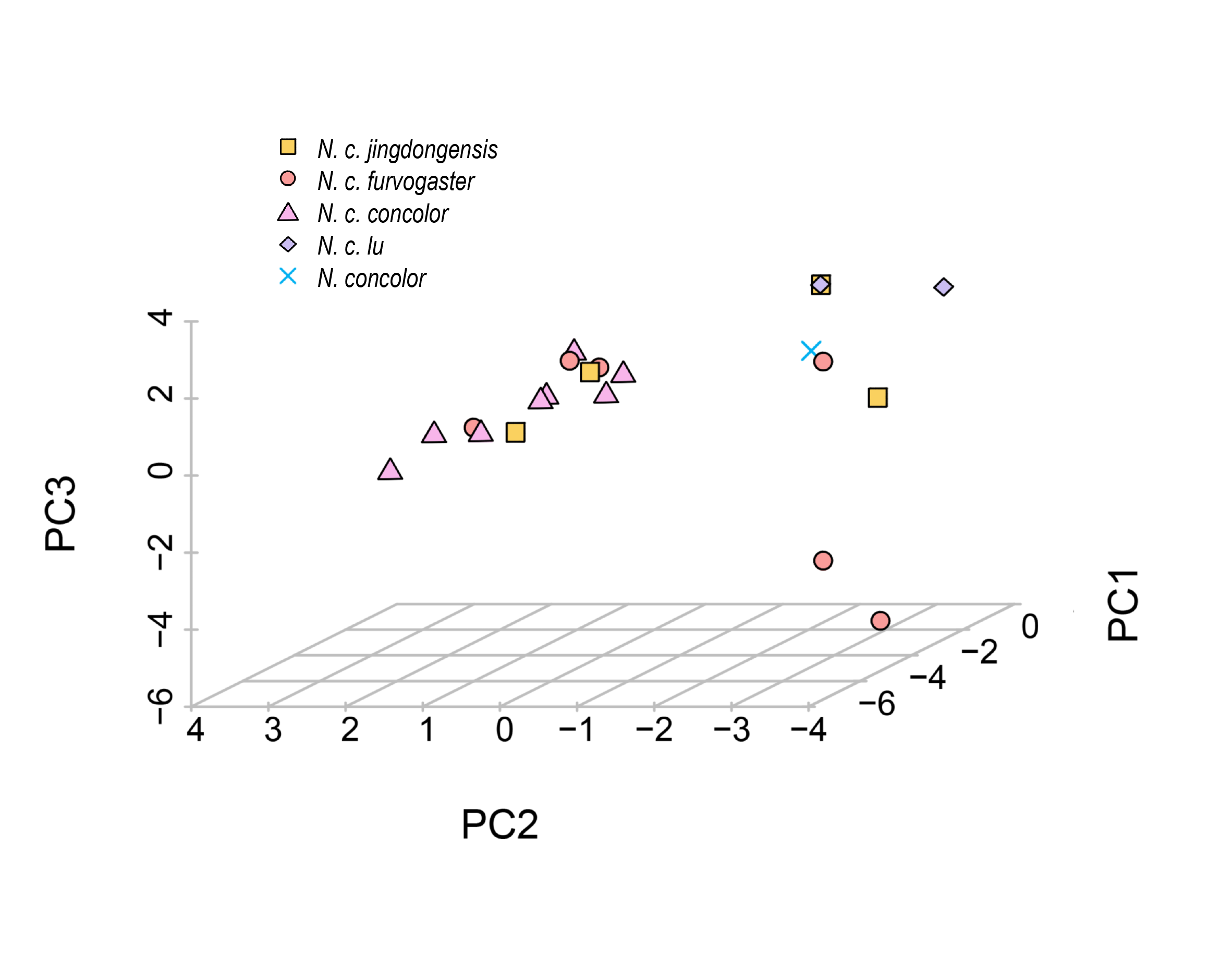


Figure S6 Principal component analysis (PCA) based on SNPs of *cytb* gene.


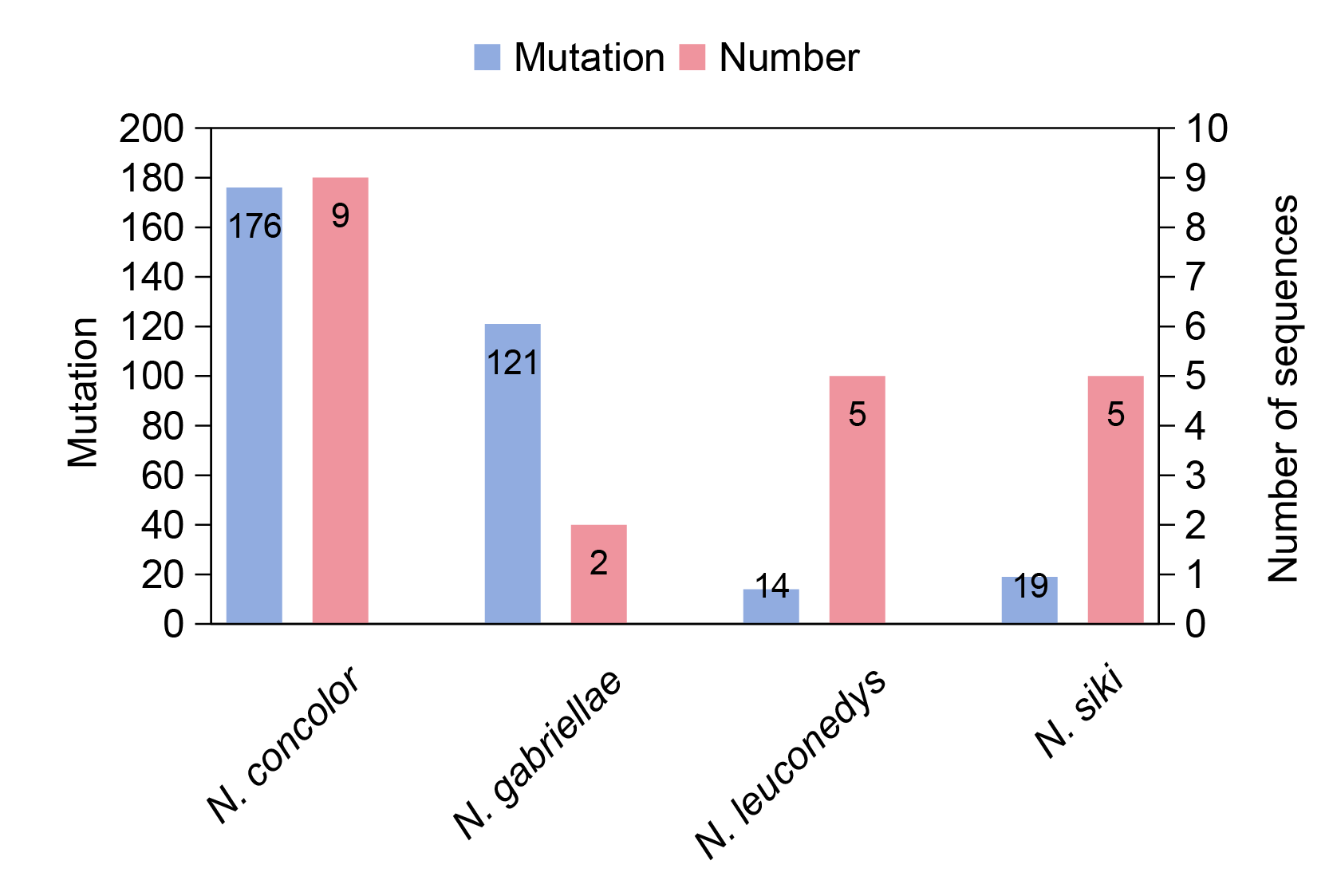


Figure S7 Number of unique mutation sites of five *Nomascus* species.


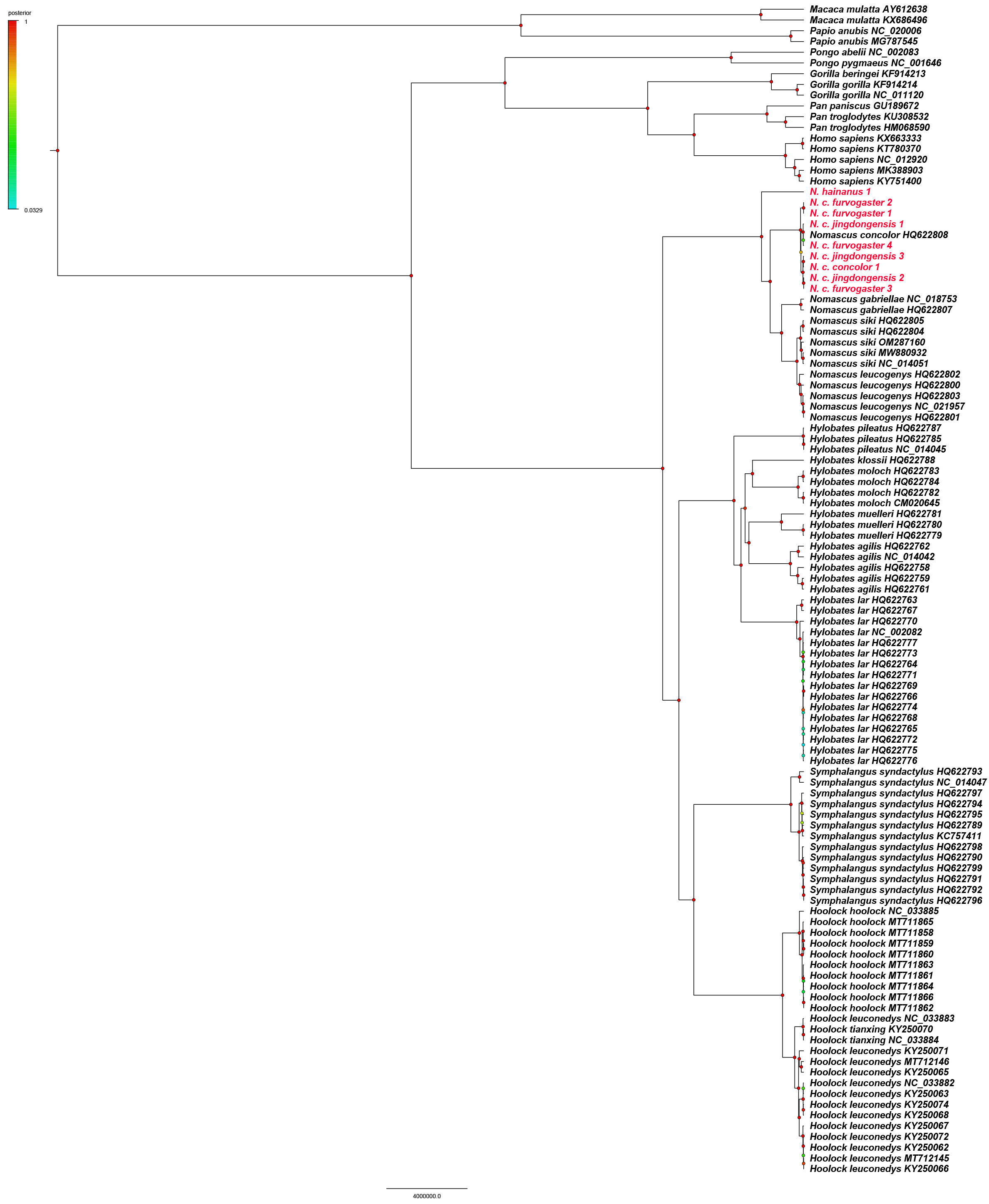


Figure S8 Bayesian phylogenetic tree based on the protein-coding sequences (CDS).


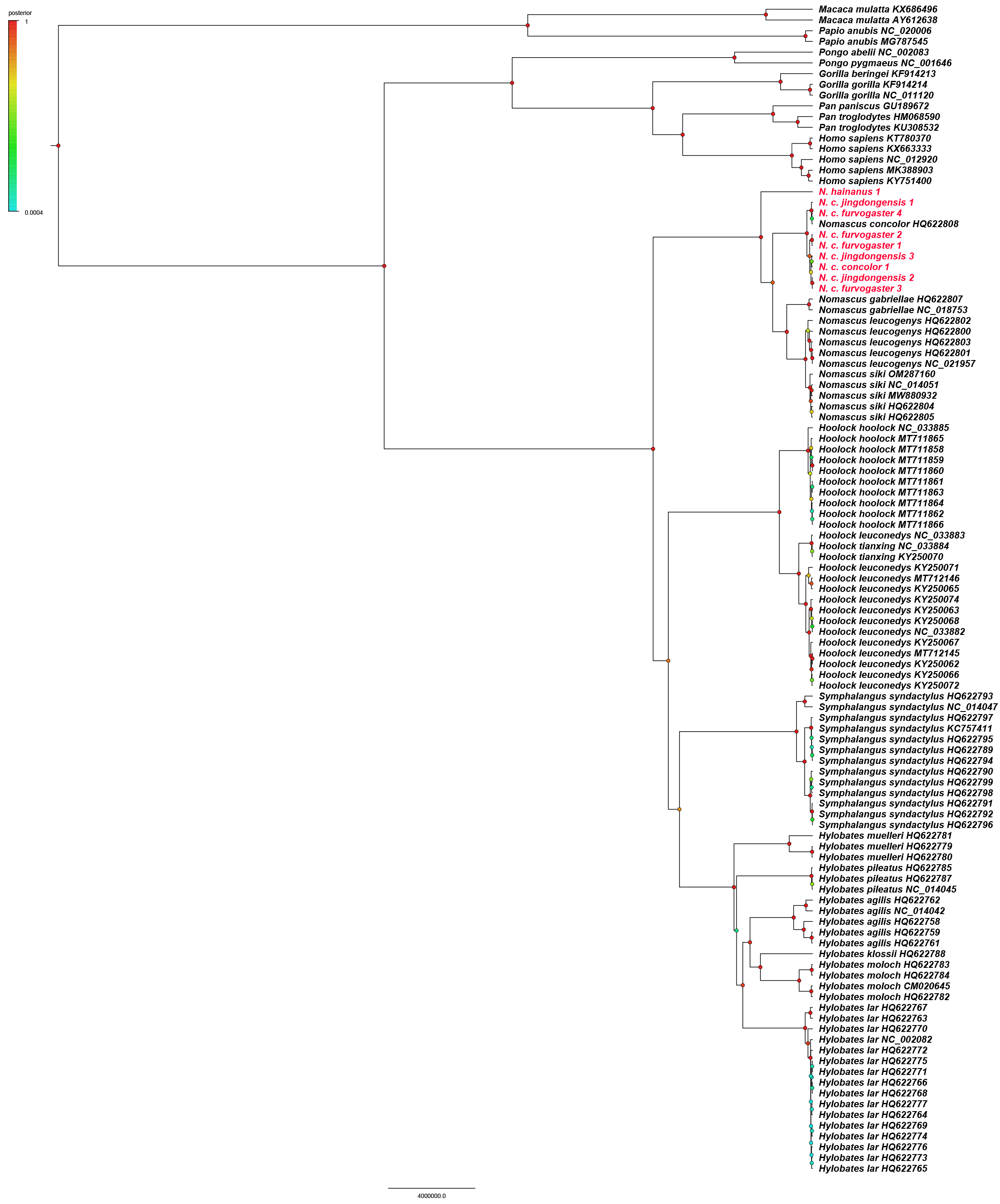


Figure S9 Bayesian phylogenetic tree based on rRNAs.


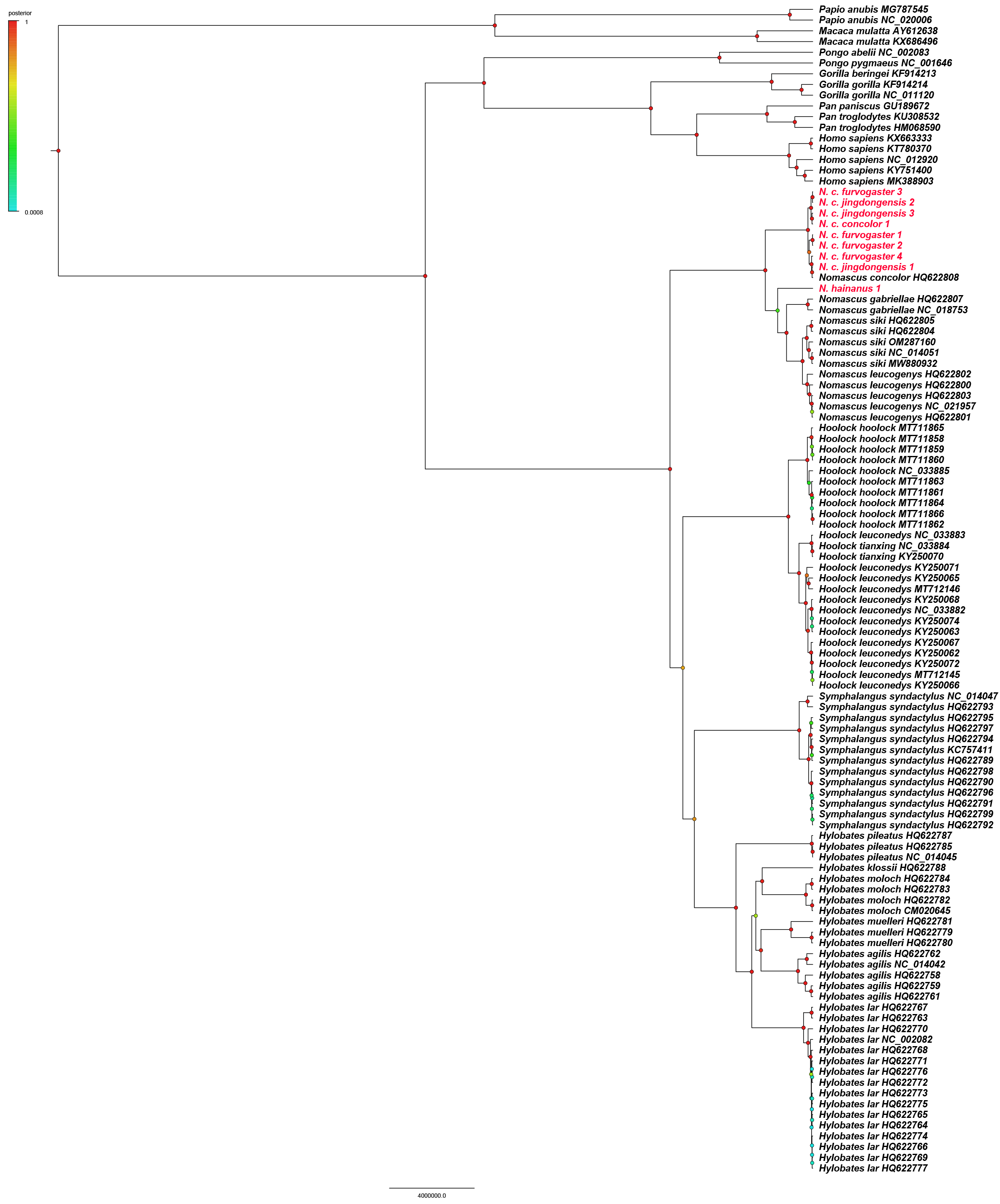


Figure S10 Bayesian phylogenetic tree based on 1^st^-2^nd^ codon.


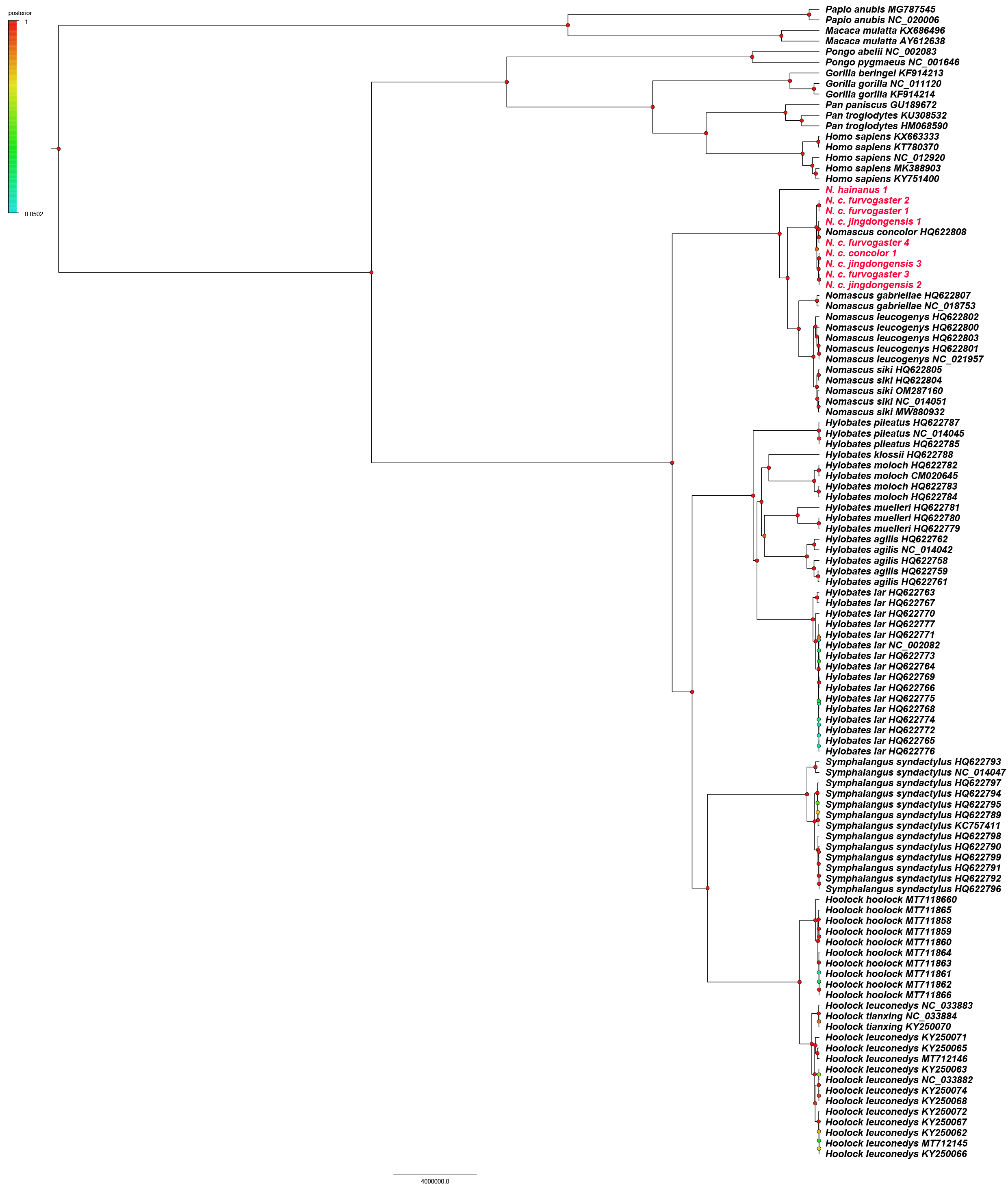


Figure S11 Bayesian phylogenetic tree based on 3^rd^ codon.


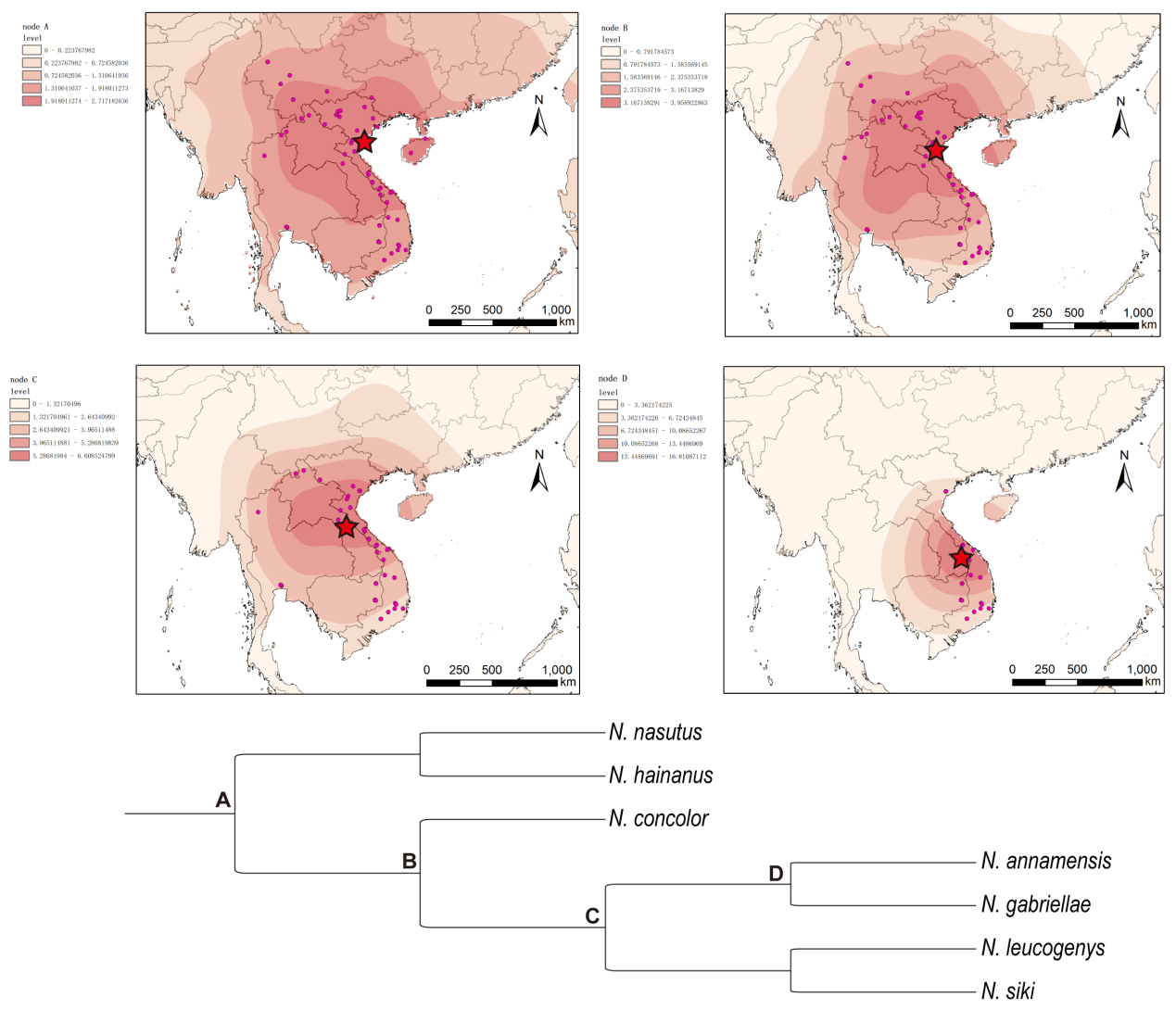


Figure S12 Putative ancestral distribution of phylogenetic nodes within *Nomascus*. The red stars indicate posterior means of potentially ancestral habitat areas inferred by BayesTraits v4.0.0. The purple dots represent the geographical positions of the individuals within the clade under the nodes (A, B, C, and D).


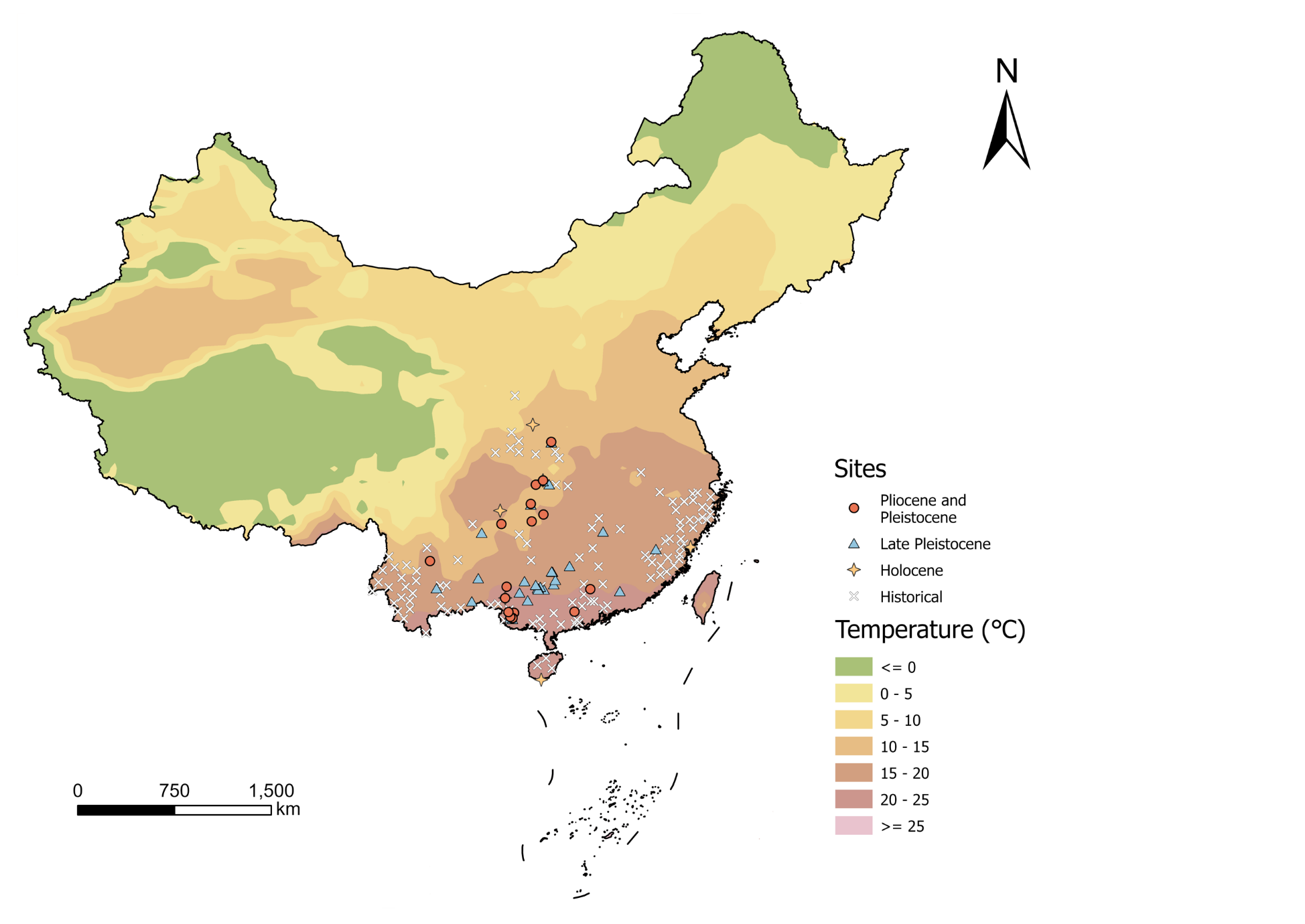


Figure S13 Fossil sites and historical distribution of gibbons in China relative to temperature.


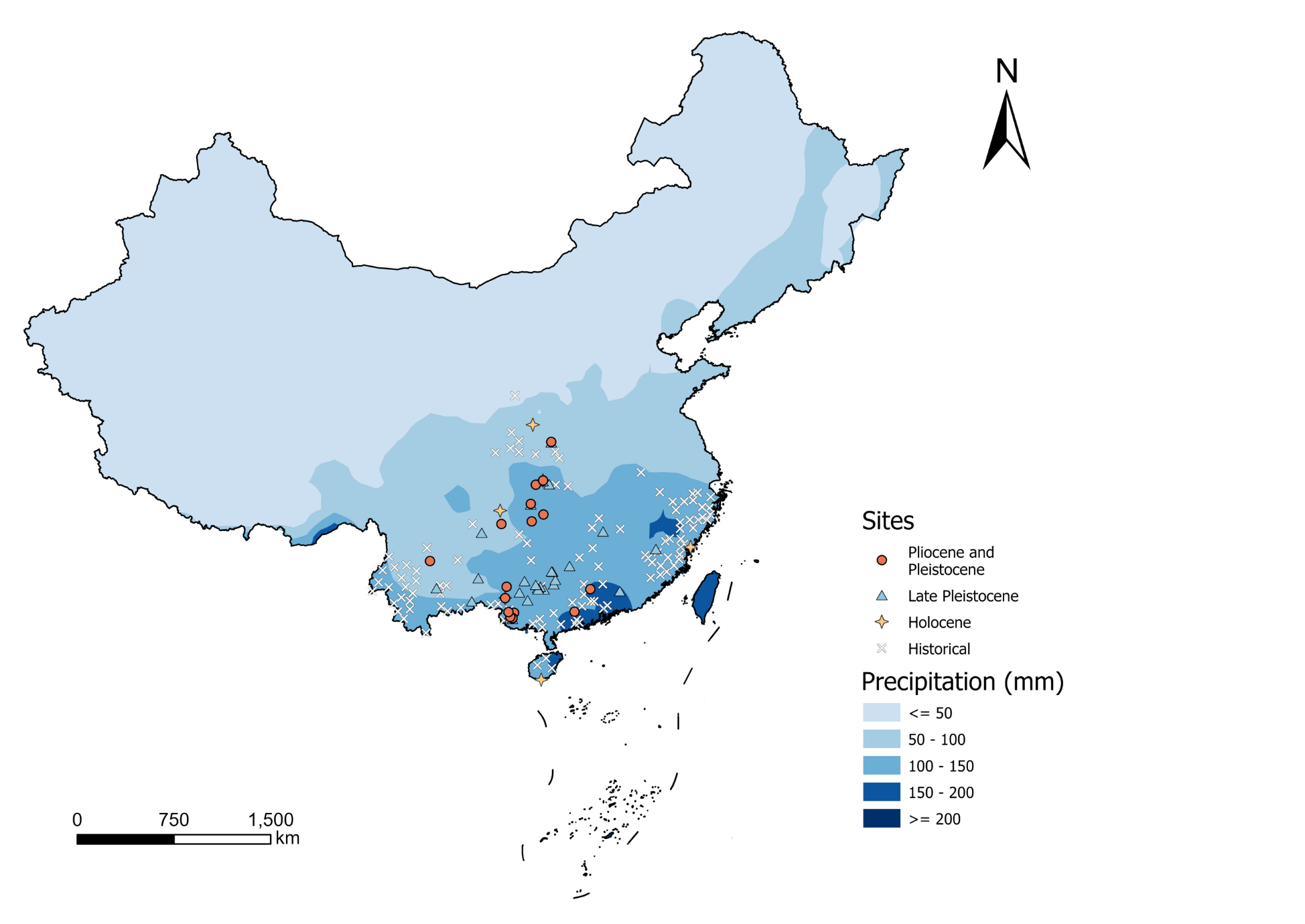


Figure S14 Fossil sites and historical distribution of gibbons in China relative to precipitation.


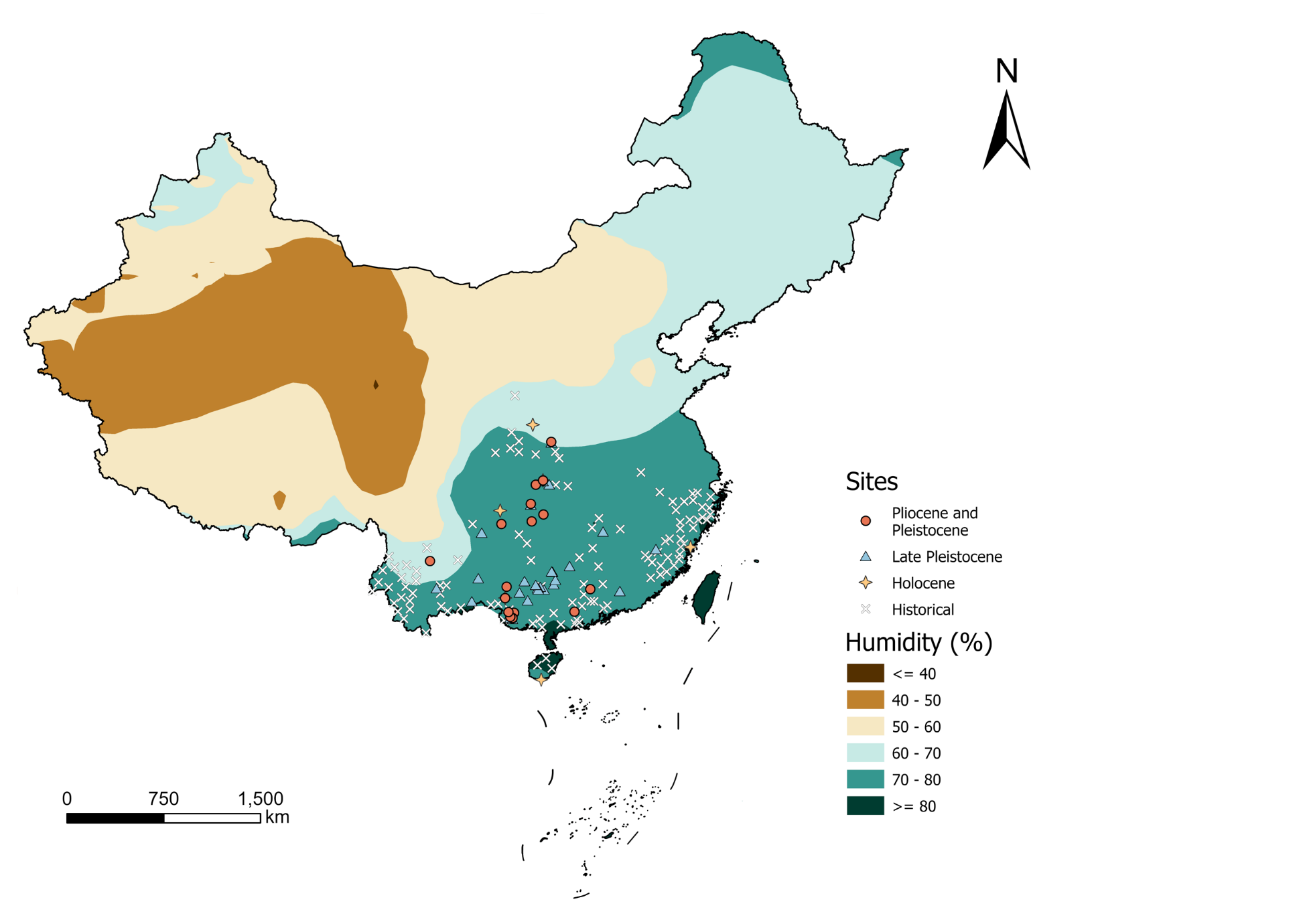


Figure S15 Fossil sites and historical distribution of gibbons in China relative to humidity. The percent of relative humidity is shown.


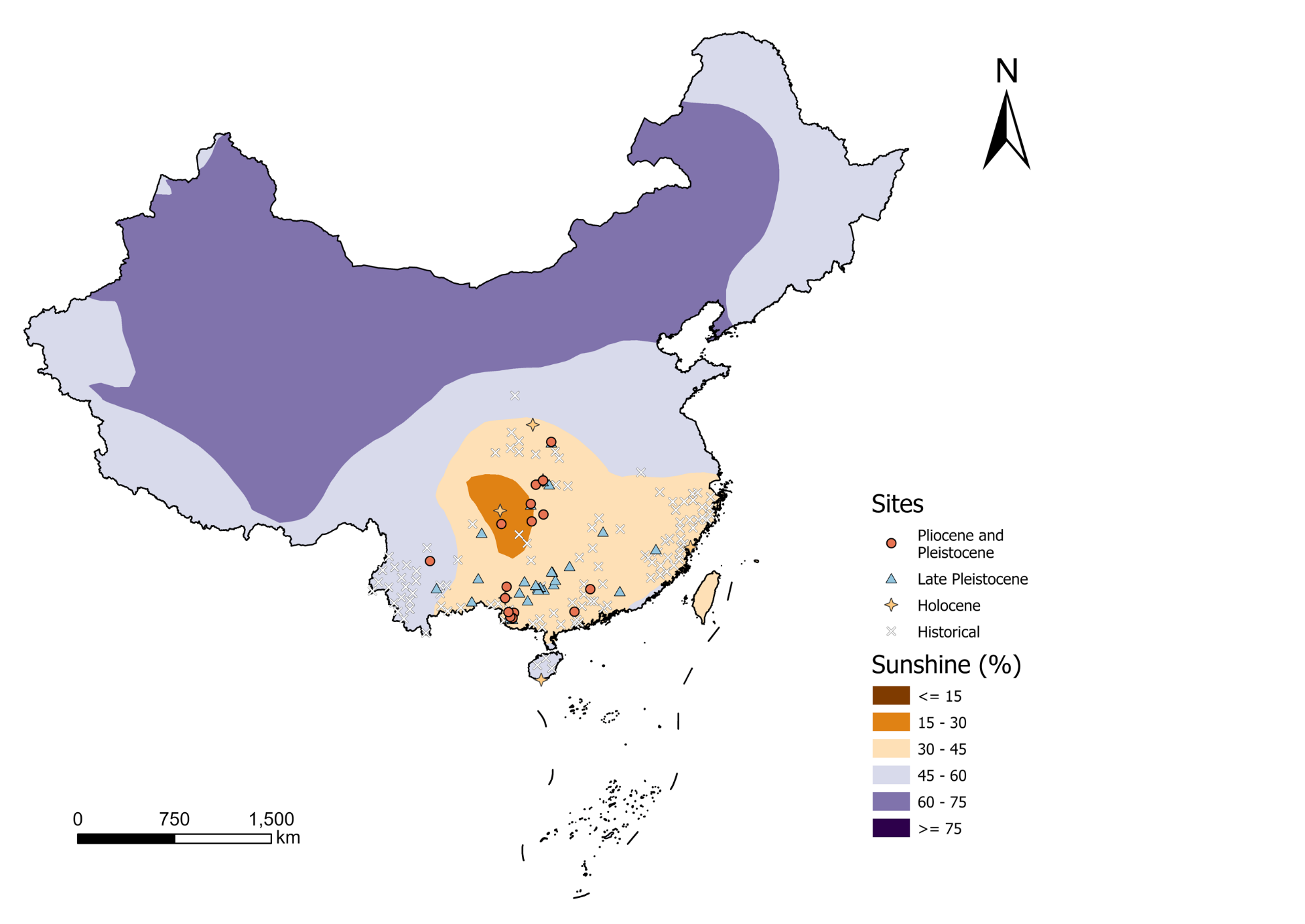


Figure S16 Fossil sites and historical distribution of gibbons in China relative to sunshine. The percent of daylength is shown.


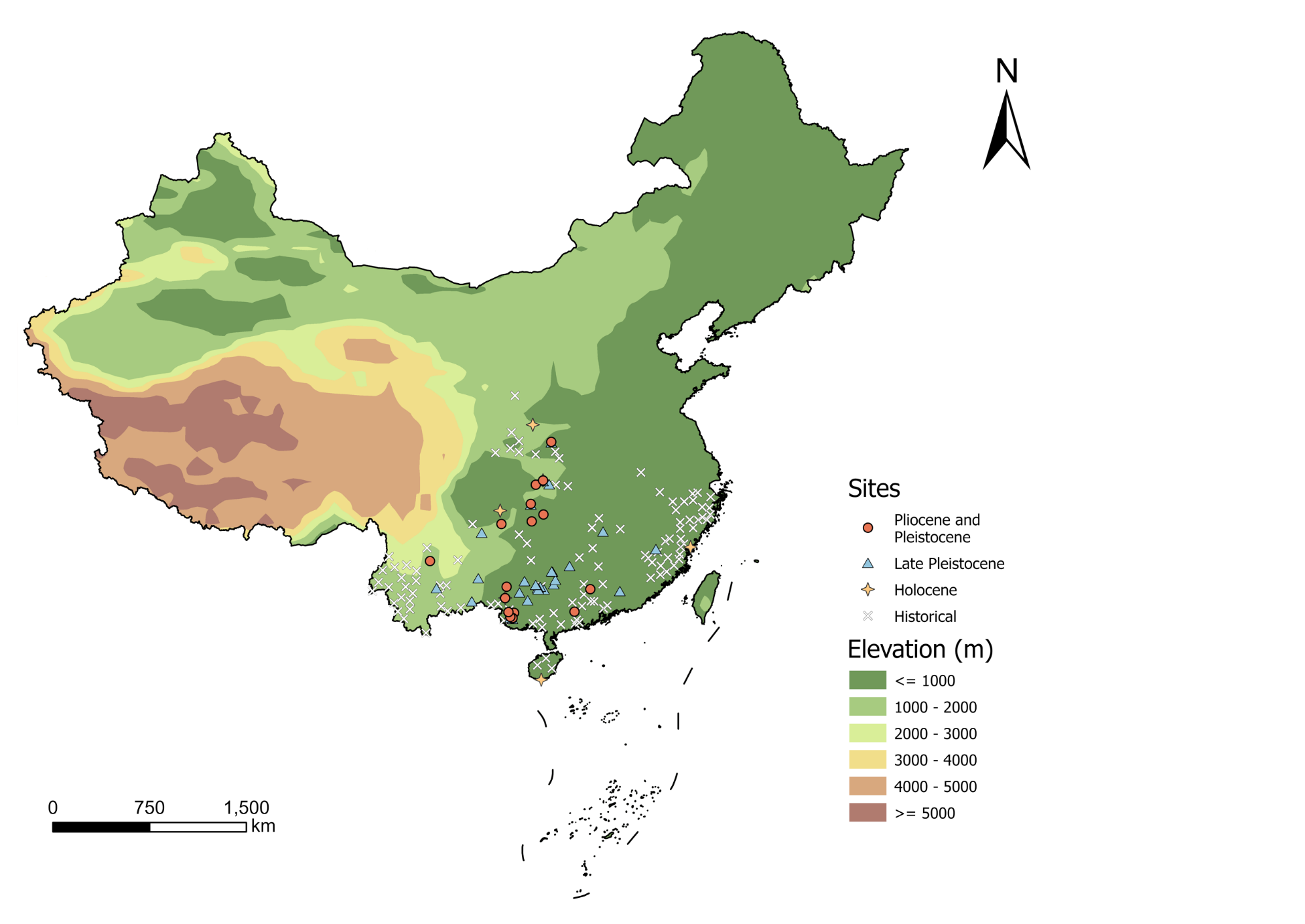


Figure S17 Fossil sites and historical distribution of gibbons in China relative to elevation.


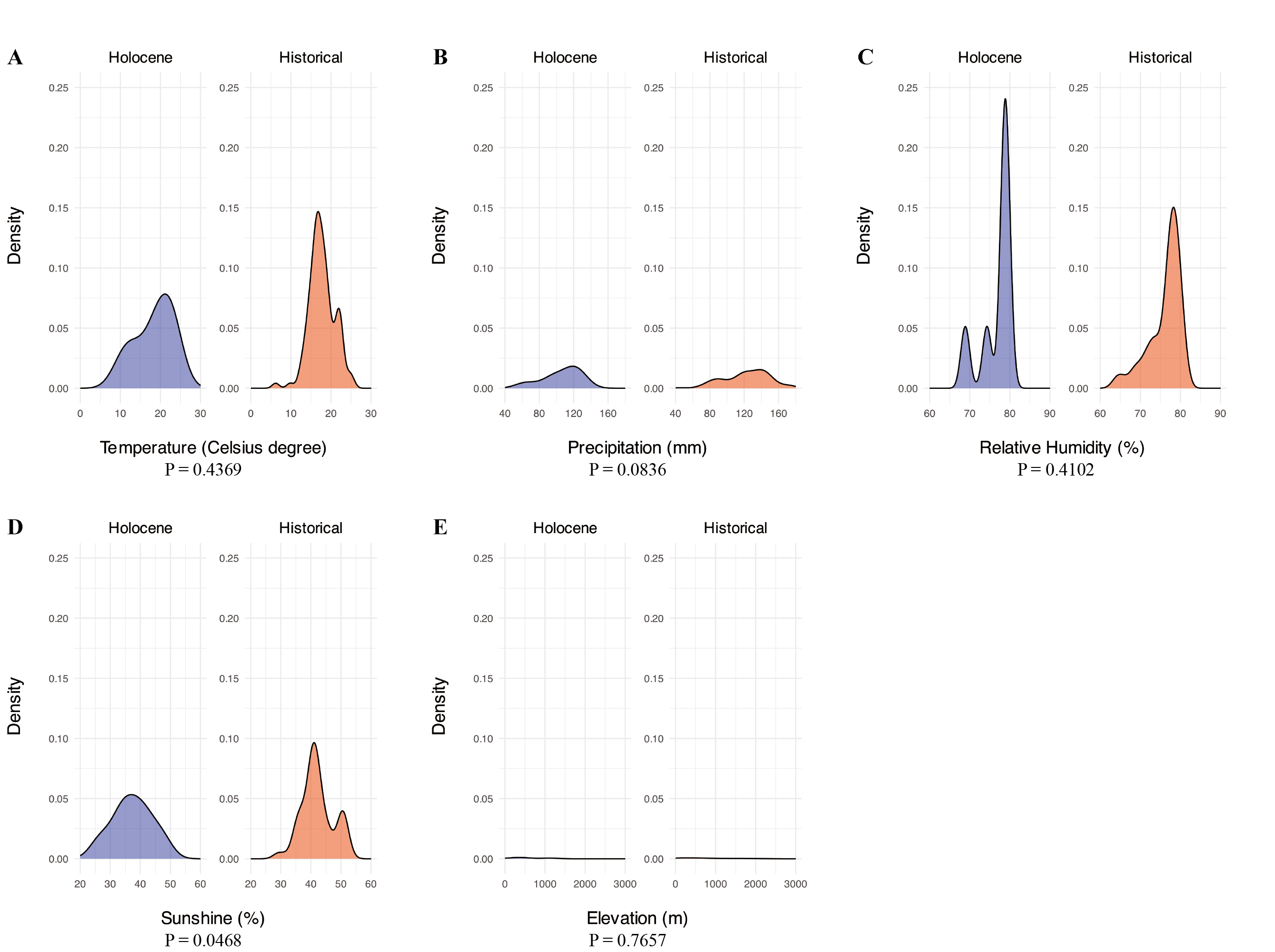


Figure S18 The distribution of climatic or geographic factor values associated with the fossil and historical records of gibbons.
