# Supplementary material for: Mitogenomes of museum specimens provide new insight into species classification and recently reduced diversity of highly endangered Nomascus gibbons
Source: Integr Zool. 2024 Jul 29;20(3):674–84. doi: 10.1111/1749-4877.12878 (PMC12046444; doi:10.1111/1749-4877.12878)
Supplement: Supplementary file 2 — Table S1 New samples sequenced for this study Table S2 Mitochondrial full‐length or cytb sequences from published papers Table S3 Comparison of estimated divergence times for different partitions Table S4 Fossil sites of gibbon family used in our study Table S5 Diversity indices calculated using mitochondrial genomes for different species in gibbon family Table S6 Comparison of D‐loop genetic diversity with other primates Table S7 Diversity indices for different sites calculated by cytb Table S8 Selection pressures on N. concolor and N. hainanus Table S9 Selection pressures on different branches of N. concolor [file INZ2-20-674-s001.docx]

**SUPPLEMENTARY MATERIALS**

**Table S1** New samples sequenced for this study

| ID | Species | Site† | Country/province | Sampling time | Amount (mg) | Concentration (ng/µL) | Raw reads | Coverage | Contamination (%) |
| --- | --- | --- | --- | --- | --- | --- | --- | --- | --- |
| *N. hainanus 1* | *Nomascus hainanus* | / | Hainan, China | / | 200 | 186 | 337268350 | 1.37 | 0.3 |
| *N. c. jingdongensis 1* | *Nomascus concolor jingdongensis* | Simao District | Yunnan, China | 1964.08.09 | 18.5 | 231.8 | 139130621 | 296.68 | 0 |
| *N. c. furvogaster 1* | *Nomascus concolor furvogaster* | Lincang | Yunnan, China | 1983.12.25 | 20.3 | 576.1 | 86940418 | 197.73 | 0 |
| *N. c. furvogaster 2* | *Nomascus concolor furvogaster* | Lincang | Yunnan, China | 1983.12.19 | 17.6 | 230.9 | 100585097 | 85.68 | 0 |
| *N. c. concolor 1* | *Nomascus concolor concolor* | Honghe Hani and Yi Autonomous Prefecture | Yunnan, China | 1972.04.30 | 6.6 | 306.8 | 141400666 | 197.57 | 0 |
| *N. c. jingdongensis 2* | *Nomascus concolor jingdongensis* | Simao District | Yunnan, China | 1957.10.14 | 21.2 | 312.6 | 196990640 | 1659.93 | 0 |
| *N. c. jingdongensis 3* | *Nomascus concolor jingdongensis* | Simao District | Yunnan, China | 1957.10.17 | 16.7 | 217.2 | 68954021 | 108.84 | 0 |
| *N. c. furvogaster 3* | *Nomascus concolor furvogaster* | / | Yunnan, China | 1982.08.00 | 7.8 | 186.3 | 113963926 | 175.17 | 0 |
| *N. c. furvogaster 4* | *Nomascus concolor furvogaster* | Southwest Yunnan | Yunnan, China | 1975.10.26 | 9.1 | 255.2 | 44195249 | 136.53 | 0 |

†The *N. hainanus* is from the Sun Yat-sen University Museum and the *N. concolor* is from the Kunming Institute of Zoology, Chinese Academy of Sciences.

**Table S2** Mitochondrial full-length or *cytb* sequences from published papers

| Accession No. | Species | Datatype | References |
| --- | --- | --- | --- |
| GU321248 | *Nomascus hainanus* | *cytb* | Thinh *et al*. 2010a |
| GU321249 | *Nomascus concolor concolor* | *cytb* | Thinh *et al*. 2010a |
| GU321250 | *Nomascus concolor concolor* | *cytb* | Thinh *et al*. 2010a |
| GU321251 | *Nomascus concolor concolor* | *cytb* | Thinh *et al*. 2010a |
| GU321252 | *Nomascus concolor concolor* | *cytb* | Thinh *et al*. 2010a |
| GU321253 | *Nomascus concolor concolor* | *cytb* | Thinh *et al*. 2010a |
| GU594997 | *Nomascus concolor concolor* | *cytb* | Thinh *et al*. 2010b |
| GU594998 | *Nomascus concolor concolor* | *cytb* | Thinh *et al*. 2010b |
| GU321254 | *Nomascus concolor furvogaster* | *cytb* | Thinh *et al.* 2010a |
| GU321255 | *Nomascus concolor furvogaster* | *cytb* | Thinh *et al*. 2010a |
| GU321256 | *Nomascus concolor jingdongensis* | *cytb* | Thinh *et al*. 2010a |
| GU321257 | *Nomascus concolor lu* | *cytb* | Thinh *et al*. 2010a |
| GU321258 | *Nomascus concolor lu* | *cytb* | Thinh *et al*. 2010a |
| HQ622808 | *Nomascus concolor* | Full-length&*cytb* | Chan *et al*. 2010 |
| MW880932 | *Nomascus siki* | Full-length&*cytb* | Unpublished |
| OM287160 | *Nomascus siki* | Full-length&*cytb* | Roos *et al*. 2022 |
| NC_014051 | *Nomascus siki* | Full-length&*cytb* | Matsudaira & Ishida 2010 |
| HQ622804 | *Nomascus siki* | Full-length&*cytb* | Chan *et al*. 2010 |
| HQ622805 | *Nomascus siki* | Full-length&*cytb* | Chan *et al*. 2010 |
| GU595005 | *Nomascus siki* | *cytb* | Thinh *et al*. 2010b |
| GU595006 | *Nomascus siki* | *cytb* | Thinh *et al*. 2010b |
| GU595007 | *Nomascus siki* | *cytb* | Thinh *et al*. 2010b |
| GU595008 | *Nomascus siki* | *cytb* | Thinh *et al*. 2010b |
| MH188412 | *Nomascus siki* | *cytb* | Nie *et al*. 2018 |
| GU321267 | *Nomascus siki* | *cytb* | Thinh *et al*. 2010a |
| GU321268 | *Nomascus siki* | *cytb* | Thinh *et al*. 2010a |
| GU321269 | *Nomascus siki* | *cytb* | Thinh *et al*. 2010a |
| GU321270 | *Nomascus siki* | *cytb* | Thinh *et al*. 2010a |
| GU321271 | *Nomascus siki* | *cytb* | Thinh *et al*. 2010a |
| GU321272 | *Nomascus siki* | *cytb* | Thinh *et al*. 2010a |
| NC_021957 | *Nomascus leucogenys* | Full-length&*cytb* | Finstermeier *et al*. 2013 |
| HQ622802 | *Nomascus leucogenys* | Full-length&*cytb* | Chan *et al*. 2010 |
| HQ622800 | *Nomascus leucogenys* | Full-length&*cytb* | Chan *et al*. 2010 |
| HQ622803 | *Nomascus leucogenys* | Full-length&*cytb* | Chan *et al*. 2010 |
| HQ622801 | *Nomascus leucogenys* | Full-length&*cytb* | Chan *et al*. 2010 |
| GU595004 | *Nomascus leucogenys* | *cytb* | Thinh *et al*. 2010b |
| GU321259 | *Nomascus leucogenys* | *cytb* | Thinh *et al*. 2010a |
| GU321260 | *Nomascus leucogenys* | *cytb* | Thinh *et al*. 2010a |
| GU321261 | *Nomascus leucogenys* | *cytb* | Thinh *et al*. 2010a |
| GU321262 | *Nomascus leucogenys* | *cytb* | Thinh *et al*. 2010a |
| GU321263 | *Nomascus leucogenys* | *cytb* | Thinh *et al*. 2010a |
| GU321264 | *Nomascus leucogenys* | *cytb* | Thinh *et al*. 2010a |
| GU321265 | *Nomascus leucogenys* | *cytb* | Thinh *et al*. 2010a |
| GU321266 | *Nomascus leucogenys* | *cytb* | Thinh *et al*. 2010a |
| GU594999 | *Nomascus leucogenys* | *cytb* | Thinh *et al*. 2010b |
| GU595000 | *Nomascus leucogenys* | *cytb* | Thinh *et al*. 2010b |
| GU595001 | *Nomascus leucogenys* | *cytb* | Thinh *et al*. 2010b |
| GU595002 | *Nomascus leucogenys* | *cytb* | Thinh *et al*. 2010b |
| GU595003 | *Nomascus leucogenys* | *cytb* | Thinh *et al*. 2010b |
| LC548051 | *Nomascus leucogenys* | *cytb* | Matsudaira & Ishida 2021b |
| LC576802 | *Nomascus leucogenys* | *cytb* | Matsudaira & Ishida 2021a |
| LC576803 | *Nomascus leucogenys* | *cytb* | Matsudaira & Ishida 2021a |
| MH188411 | *Nomascus leucogenys* | *cytb* | Nie *et al*. 2018 |
| MH188414 | *Nomascus leucogenys* | *cytb* | Nie *et al*. 2018 |
| MH188409 | *Nomascus leucogenys x Nomascus siki* | *cytb* | Nie *et al*. 2018 |
| HQ622807 | *Nomascus gabriellae* | Full-length&*cytb* | Chan *et al*. 2010 |
| NC_018753 | *Nomascus gabriellae* | Full-length&*cytb* | Chan *et al*. 2010 |
| GU321273 | *Nomascus gabriellae* | *cytb* | Thinh *et al*. 2010a |
| GU321274 | *Nomascus gabriellae* | *cytb* | Thinh *et al*. 2010a |
| GU321275 | *Nomascus gabriellae* | *cytb* | Thinh *et al*. 2010a |
| GU321276 | *Nomascus gabriellae* | *cytb* | Thinh *et al*. 2010a |
| GU321277 | *Nomascus gabriellae* | *cytb* | Thinh *et al*. 2010a |
| GU321278 | *Nomascus gabriellae* | *cytb* | Thinh *et al*. 2010a |
| GU321279 | *Nomascus gabriellae* | *cytb* | Thinh *et al*. 2010a |
| GU321280 | *Nomascus gabriellae* | *cytb* | Thinh *et al*. 2010a |
| GU321281 | *Nomascus gabriellae* | *cytb* | Thinh *et al*. 2010a |
| GU595016 | *Nomascus gabriellae* | *cytb* | Thinh *et al*. 2010b |
| GU595017 | *Nomascus gabriellae* | *cytb* | Thinh *et al*. 2010b |
| GU595018 | *Nomascus gabriellae* | *cytb* | Thinh *et al*. 2010b |
| GU595019 | *Nomascus gabriellae* | *cytb* | Thinh *et al*. 2010b |
| GU595020 | *Nomascus gabriellae* | *cytb* | Thinh *et al*. 2010b |
| GU595021 | *Nomascus gabriellae* | *cytb* | Thinh *et al*. 2010b |
| GU595022 | *Nomascus gabriellae* | *cytb* | Thinh *et al*. 2010b |
| MH188410 | *Nomascus gabriellae* | *cytb* | Nie *et al*. 2018 |
| MH188415 | *Nomascus gabriellae* | *cytb* | Nie *et al*. 2018 |
| MH188416 | *Nomascus gabriellae* | *cytb* | Nie *et al*. 2018 |
| MN615763 | *Nomascus gabriellae* | *cytb* | Leroux *et al*. 2020 |
| MH188408 | *Nomascus gabriellae x Nomascus siki* | *cytb* | Nie *et al*. 2018 |
| MH188413 | *Nomascus gabriellae x Nomascus siki* | *cytb* | Nie *et al*. 2018 |
| LC576813 | *Nomascus leucogenys x Nomascus gabriellae* | *cytb* | Matsudaira & Ishida 2021a |
| GU595009 | *Nomascus annamensis* | *cytb* | Thinh *et al*. 2010b |
| GU595010 | *Nomascus annamensis* | *cytb* | Thinh *et al*. 2010b |
| GU595011 | *Nomascus annamensis* | *cytb* | Thinh *et al*. 2010b |
| GU595012 | *Nomascus annamensis* | *cytb* | Thinh *et al*. 2010b |
| GU595013 | *Nomascus annamensis* | *cytb* | Thinh *et al*. 2010b |
| GU595014 | *Nomascus annamensis* | *cytb* | Thinh *et al*. 2010b |
| GU595015 | *Nomascus annamensis* | *cytb* | Thinh *et al*. 2010b |
| MN615755 | *Nomascus annamensis* | *cytb* | Leroux *et al*. 2020 |
| MN615769 | *Nomascus annamensis* | *cytb* | Leroux *et al*. 2020 |
| MN615772 | *Nomascus annamensis* | *cytb* | Leroux *et al*. 2020 |
| MN615774 | *Nomascus annamensis* | *cytb* | Leroux *et al*. 2020 |
| GU321245 | *Nomascus nasutus* | *cytb* | Thinh *et al*. 2010a |
| GU321246 | *Nomascus nasutus* | *cytb* | Thinh *et al*. 2010a |
| GU321247 | *Nomascus nasutus* | *cytb* | Thinh *et al*. 2010a |
| GU594996 | *Nomascus nasutus* | *cytb* | Thinh *et al*. 2010b |
| MH188417 | *Nomascus cf. leucogenys SSH-2018* | *cytb* | Nie *et al*. 2018 |
| MT711866 | *Hoolock hoolock* | Full-length&*cytb* | Unpublished |
| MT711865 | *Hoolock hoolock* | Full-length&*cytb* | Unpublished |
| MT711864 | *Hoolock hoolock* | Full-length&*cytb* | Unpublished |
| MT711863 | *Hoolock hoolock* | Full-length&*cytb* | Unpublished |
| MT711862 | *Hoolock hoolock* | Full-length&*cytb* | Unpublished |
| MT711861 | *Hoolock hoolock* | Full-length&*cytb* | Unpublished |
| MT711860 | *Hoolock hoolock* | Full-length&*cytb* | Unpublished |
| MT711859 | *Hoolock hoolock* | Full-length&*cytb* | Unpublished |
| MT711858 | *Hoolock hoolock* | Full-length&*cytb* | Unpublished |
| NC_033885 | *Hoolock hoolock* | Full-length | Fan *et al*. 2017 |
| GU321287 | *Hoolock hoolock* | *cytb* | Fan *et al*. 2017 |
| GU321286 | *hoolock hoolock* | *cytb* | Thinh *et al*. 2010a |
| MW242797 | *Hoolock hoolock* | *cytb* | Unpublished |
| MW242798 | *Hoolock hoolock* | *cytb* | Unpublished |
| MW242799 | *Hoolock hoolock* | *cytb* | Unpublished |
| MW242800 | *Hoolock hoolock* | *cytb* | Unpublished |
| MW242801 | *Hoolock hoolock* | *cytb* | Unpublished |
| MW242802 | *Hoolock hoolock* | *cytb* | Unpublished |
| MW242803 | *Hoolock hoolock* | *cytb* | Unpublished |
| MW242804 | *Hoolock hoolock* | *cytb* | Unpublished |
| MW242805 | *Hoolock hoolock* | *cytb* | Unpublished |
| MW242806 | *Hoolock hoolock* | *cytb* | Unpublished |
| MW242807 | *Hoolock hoolock* | *cytb* | Unpublished |
| MW242808 | *Hoolock hoolock* | *cytb* | Unpublished |
| MW242809 | *Hoolock hoolock* | *cytb* | Unpublished |
| MT712146 | *Hoolock leuconedys* | Full-length&*cytb* | Unpublished |
| MT712145 | *Hoolock leuconedys* | Full-length&*cytb* | Unpublished |
| NC_033882 | *Hoolock leuconedys* | Full-length&*cytb* | Fan *et al*. 2017 |
| GU321290 | *Hoolock leuconedys* | *cytb* | Fan *et al*. 2017 |
| KY250074 | *Hoolock leuconedys* | Full-length | Fan *et al*. 2017 |
| KY250071 | *Hoolock leuconedys* | Full-length&*cytb* | Fan *et al*. 2017 |
| KY250068 | *Hoolock leuconedys* | Full-length&*cytb* | Fan *et al*. 2017 |
| KY250067 | *Hoolock leuconedys* | Full-length&*cytb* | Fan *et al*. 2017 |
| KY250066 | *Hoolock leuconedys* | Full-length&*cytb* | Fan *et al*. 2017 |
| KY250065 | *Hoolock leuconedys* | Full-length&*cytb* | Fan *et al*. 2017 |
| KY250063 | *Hoolock leuconedys* | Full-length&*cytb* | Fan *et al*. 2017 |
| KY250062 | *Hoolock leuconedys* | Full-length&*cytb* | Fan *et al*. 2017 |
| KY250072 | *Hoolock leuconedys x Hoolock tianxing* | Full-length&*cytb* | Fan *et al*. 2017 |
| GU321288 | *Hoolock leuconedys* | *cytb* | Thinh *et al*. 2010a |
| GU321289 | *Hoolock leuconedys* | *cytb* | Thinh *et al*. 2010a |
| KY250058 | *Hoolock leuconedys* | *cytb* | Fan *et al*. 2017 |
| KY250060 | *Hoolock leuconedys* | *cytb* | Fan *et al*. 2017 |
| LC500934 | *Hoolock leuconedys* | *cytb* | Unpublished |
| LC500935 | *Hoolock leuconedys* | *cytb* | Unpublished |
| LC500936 | *Hoolock leuconedys* | *cytb* | Unpublished |
| LC500937 | *Hoolock leuconedys* | *cytb* | Unpublished |
| LC500938 | *Hoolock leuconedys* | *cytb* | Unpublished |
| LC500939 | *Hoolock leuconedys* | *cytb* | Unpublished |
| LC500940 | *Hoolock leuconedys* | *cytb* | Unpublished |
| LC500941 | *Hoolock leuconedys* | *cytb* | Unpublished |
| LC500943 | *Hoolock leuconedys* | *cytb* | Unpublished |
| NC_033884 | *Hoolock tianxing* | Full-length&*cytb* | Fan *et al*. 2017 |
| KY250070 | *Hoolock tianxing* | Full-length&*cytb* | Fan *et al*. 2017 |
| NC_033883 | *Hoolock leuconedys x Hoolock tianxing* | Full-length&*cytb* | Fan *et al*. 2017 |
| KY250059 | *Hoolock tianxing* | *cytb* | Fan *et al*. 2017 |
| LC500942 | *Hoolock tianxing* | *cytb* | Unpublished |
| NC_014042 | *Hylobates agilis* | Full-length&*cytb* | Matsudaira &Ishida 2010 |
| HQ622758 | *Hylobates agilis* | Full-length&*cytb* | Chan *et al*. 2010 |
| HQ622762 | *Hylobates agilis* | Full-length&*cytb* | Chan *et al*. 2010 |
| HQ622759 | *Hylobates agilis* | Full-length&*cytb* | Chan *et al*. 2010 |
| HQ622761 | *Hylobates agilis* | Full-length&*cytb* | Chan *et al*. 2010 |
| AJ010583 | *Hylobates agilis* | *cytb* | Unpublished |
| GU321298 | *Hylobates agilis agilis* | *cytb* | Thinh *et al*. 2010a |
| GU321299 | *Hylobates agilis agilis* | *cytb* | Thinh *et al*. 2010a |
| GU321300 | *Hylobates agilis agilis* | *cytb* | Thinh *et al*. 2010a |
| GU321301 | *Hylobates agilis agilis* | *cytb* | Thinh *et al*. 2010a |
| GU321302 | *Hylobates agilis agilis* | *cytb* | Thinh *et al*. 2010a |
| GU321303 | *Hylobates agilis agilis* | *cytb* | Thinh *et al*. 2010a |
| GU321304 | *Hylobates agilis unko* | *cytb* | Thinh *et al*. 2010a |
| GU321305 | *Hylobates agilis unko* | *cytb* | Thinh *et al*. 2010a |
| LC576757 | *Hylobates agilis* | *cytb* | Matsudaira & Ishida 2021a |
| LC576758 | *Hylobates agilis* | *cytb* | Matsudaira & Ishida 2021a |
| LC576759 | *Hylobates agilis* | *cytb* | Matsudaira & Ishida 2021a |
| LC576760 | *Hylobates agilis* | *cytb* | Matsudaira & Ishida 2021a |
| LC576761 | *Hylobates agilis* | *cytb* | Matsudaira & Ishida 2021a |
| LC576762 | *Hylobates agilis* | *cytb* | Matsudaira & Ishida 2021a |
| LC576763 | *Hylobates agilis* | *cytb* | Matsudaira & Ishida 2021a |
| LC576764 | *Hylobates agilis* | *cytb* | Matsudaira & Ishida 2021a |
| LC576814 | *Hylobates agilis x Hylobates albibarbis* | *cytb* | Matsudaira & Ishida 2021a |
| GU321306 | *Hylobates albibarbis* | *cytb* | Thinh *et al*. 2010a |
| GU321307 | *Hylobates albibarbis* | *cytb* | Thinh *et al*. 2010a |
| LC548017 | *Hylobates albibarbis* | *cytb* | Matsudaira & Ishida 2021b |
| LC548018 | *Hylobates albibarbis* | *cytb* | Matsudaira & Ishida 2021b |
| LC576765 | *Hylobates albibarbis* | *cytb* | Matsudaira & Ishida 2021b |
| LC576766 | *Hylobates albibarbis* | *cytb* | Matsudaira & Ishida 2021b |
| LC576767 | *Hylobates albibarbis* | *cytb* | Matsudaira & Ishida 2021b |
| LC576768 | *Hylobates albibarbis* | *cytb* | Matsudaira & Ishida 2021b |
| HQ622776 | *Hylobates lar* | Full-length&*cytb* | Chan *et al*. 2010 |
| HQ622775 | *Hylobates lar* | Full-length&*cytb* | Chan *et al*. 2010 |
| HQ622771 | *Hylobates lar* | Full-length&*cytb* | Chan *et al*. 2010 |
| HQ622766 | *Hylobates lar* | Full-length&*cytb* | Chan *et al*. 2010 |
| NC_002082 | *Hylobates lar* | Full-length&*cytb* | Arnason *et al*. 1996 |
| HQ622774 | *Hylobates lar* | Full-length&*cytb* | Chan *et al*. 2010 |
| HQ622770 | *Hylobates lar* | Full-length&*cytb* | Chan *et al*. 2010 |
| HQ622772 | *Hylobates lar* | Full-length&*cytb* | Chan *et al*. 2010 |
| HQ622763 | *Hylobates lar* | Full-length&*cytb* | Chan *et al*. 2010 |
| HQ622764 | *Hylobates lar* | Full-length&*cytb* | Chan *et al*. 2010 |
| HQ622773 | *Hylobates lar* | Full-length&*cytb* | Chan *et al*. 2010 |
| HQ622777 | *Hylobates lar* | Full-length&*cytb* | Chan *et al*. 2010 |
| HQ622765 | *Hylobates lar* | Full-length&*cytb* | Chan *et al*. 2010 |
| HQ622768 | *Hylobates lar* | Full-length&*cytb* | Chan *et al*. 2010 |
| HQ622769 | *Hylobates lar* | Full-length&*cytb* | Chan *et al*. 2010 |
| HQ622767 | *Hylobates lar* | Full-length&*cytb* | Chan *et al*. 2010 |
| GU321319 | *Hylobates lar* | *cytb* | Thinh *et al*. 2010a |
| GU321320 | *Hylobates lar* | *cytb* | Thinh *et al*. 2010a |
| GU321321 | *Hylobates lar* | *cytb* | Thinh *et al*. 2010a |
| GU321322 | *Hylobates lar* | *cytb* | Thinh *et al*. 2010a |
| GU321323 | *Hylobates lar* | *cytb* | Thinh *et al*. 2010a |
| GU321324 | *Hylobates lar* | *cytb* | Thinh *et al*. 2010a |
| GU321325 | *Hylobates lar carpenteri* | *cytb* | Thinh *et al*. 2010a |
| GU321326 | *Hylobates lar entelloides* | *cytb* | Thinh *et al*. 2010a |
| GU321327 | *Hylobates lar entelloides* | *cytb* | Thinh *et al*. 2010a |
| GU321328 | *Hylobates lar lar* | *cytb* | Thinh *et al*. 2010a |
| GU321329 | *Hylobates lar vestitus* | *cytb* | Thinh *et al*. 2010a |
| LC548019 | *Hylobates lar* | *cytb* | Matsudaira & Ishida 2021b |
| LC548020 | *Hylobates lar* | *cytb* | Matsudaira & Ishida 2021b |
| LC548021 | *Hylobates lar* | *cytb* | Matsudaira & Ishida 2021b |
| LC548022 | *Hylobates lar* | *cytb* | Matsudaira & Ishida 2021b |
| LC548023 | *Hylobates lar* | *cytb* | Matsudaira & Ishida 2021b |
| LC548024 | *Hylobates lar* | *cytb* | Matsudaira & Ishida 2021b |
| LC548025 | *Hylobates lar* | *cytb* | Matsudaira & Ishida 2021b |
| LC548026 | *Hylobates lar* | *cytb* | Matsudaira & Ishida 2021b |
| LC548027 | *Hylobates lar* | *cytb* | Matsudaira & Ishida 2021b |
| LC548028 | *Hylobates lar* | *cytb* | Matsudaira & Ishida 2021b |
| LC548029 | *Hylobates lar* | *cytb* | Matsudaira & Ishida 2021b |
| LC548030 | *Hylobates lar* | *cytb* | Matsudaira & Ishida 2021b |
| LC548031 | *Hylobates lar* | *cytb* | Matsudaira & Ishida 2021b |
| LC548032 | *Hylobates lar* | *cytb* | Matsudaira & Ishida 2021b |
| LC548033 | *Hylobates lar* | *cytb* | Matsudaira & Ishida 2021b |
| LC548034 | *Hylobates lar* | *cytb* | Matsudaira & Ishida 2021b |
| LC548035 | *Hylobates lar* | *cytb* | Matsudaira & Ishida 2021b |
| LC548036 | *Hylobates lar* | *cytb* | Matsudaira & Ishida 2021b |
| LC548037 | *Hylobates lar* | *cytb* | Matsudaira & Ishida 2021b |
| LC548038 | *Hylobates lar* | *cytb* | Matsudaira & Ishida 2021b |
| LC576769 | *Hylobates lar* | *cytb* | Matsudaira & Ishida 2021a |
| LC576770 | *Hylobates lar* | *cytb* | Matsudaira & Ishida 2021a |
| LC576771 | *Hylobates lar* | *cytb* | Matsudaira & Ishida 2021a |
| LC576772 | *Hylobates lar* | *cytb* | Matsudaira & Ishida 2021a |
| LC576773 | *Hylobates lar* | *cytb* | Matsudaira & Ishida 2021a |
| LC576774 | *Hylobates lar* | *cytb* | Matsudaira & Ishida 2021a |
| LC576775 | *Hylobates lar* | *cytb* | Matsudaira & Ishida 2021a |
| LC576776 | *Hylobates lar* | *cytb* | Matsudaira & Ishida 2021a |
| LC576777 | *Hylobates lar* | *cytb* | Matsudaira & Ishida 2021a |
| LC576778 | *Hylobates lar* | *cytb* | Matsudaira & Ishida 2021a |
| LC576779 | *Hylobates lar* | *cytb* | Matsudaira & Ishida 2021a |
| LC576780 | *Hylobates lar* | *cytb* | Matsudaira & Ishida 2021a |
| LC576781 | *Hylobates lar* | *cytb* | Matsudaira & Ishida 2021a |
| LC576782 | *Hylobates lar* | *cytb* | Matsudaira & Ishida 2021a |
| LC576783 | *Hylobates lar* | *cytb* | Matsudaira & Ishida 2021a |
| CM020645 | *Hylobates moloch* | Full-length&*cytb* | Unpublished |
| HQ622782 | *Hylobates moloch* | Full-length&*cytb* | Chan *et al*. 2010 |
| HQ622783 | *Hylobates moloch* | Full-length&*cytb* | Chan *et al*. 2010 |
| HQ622784 | *Hylobates moloch* | Full-length&*cytb* | Chan *et al*. 2010 |
| AJ010580 | *Hylobates moloch* | *cytb* | Unpublished |
| GU321295 | *Hylobates moloch* | *cytb* | Thinh *et al*. 2010a |
| GU321296 | *Hylobates moloch* | *cytb* | Thinh *et al*. 2010a |
| GU321297 | *Hylobates moloch* | *cytb* | Thinh *et al*. 2010a |
| NC_014045 | *Hylobates pileatus* | Full-length&*cytb* | Matsudaira & Ishida 2010 |
| HQ622787 | *Hylobates pileatus* | Full-length&*cytb* | Chan *et al*. 2010 |
| HQ622785 | *Hylobates pileatus* | Full-length&*cytb* | Chan *et al*. 2010 |
| AJ010582 | *Hylobates pileatus* | *cytb* | Unpublished |
| GU321291 | *Hylobates pileatus* | *cytb* | Thinh *et al*. 2010a |
| GU321292 | *Hylobates pileatus* | *cytb* | Thinh *et al*. 2010a |
| GU321293 | *Hylobates pileatus* | *cytb* | Thinh *et al*. 2010a |
| GU321294 | *Hylobates pileatus* | *cytb* | Thinh *et al*. 2010a |
| LC548041 | *Hylobates pileatus* | *cytb* | Matsudaira & Ishida 2021b |
| LC548042 | *Hylobates pileatus* | *cytb* | Matsudaira & Ishida 2021b |
| LC548043 | *Hylobates pileatus* | *cytb* | Matsudaira & Ishida 2021b |
| LC548044 | *Hylobates pileatus* | *cytb* | Matsudaira & Ishida 2021b |
| LC548045 | *Hylobates pileatus* | *cytb* | Matsudaira & Ishida 2021b |
| LC548046 | *Hylobates pileatus* | *cytb* | Matsudaira & Ishida 2021b |
| LC548047 | *Hylobates pileatus* | *cytb* | Matsudaira & Ishida 2021b |
| LC548048 | *Hylobates pileatus* | *cytb* | Matsudaira & Ishida 2021b |
| LC548049 | *Hylobates pileatus* | *cytb* | Matsudaira & Ishida 2021b |
| LC548050 | *Hylobates pileatus* | *cytb* | Matsudaira & Ishida 2021b |
| LC576792 | *Hylobates pileatus* | *cytb* | Matsudaira & Ishida 2021a |
| LC576793 | *Hylobates pileatus* | *cytb* | Matsudaira & Ishida 2021a |
| LC576794 | *Hylobates pileatus* | *cytb* | Matsudaira & Ishida 2021a |
| LC576795 | *Hylobates pileatus* | *cytb* | Matsudaira & Ishida 2021a |
| LC576796 | *Hylobates pileatus* | *cytb* | Matsudaira & Ishida 2021a |
| LC576797 | *Hylobates pileatus* | *cytb* | Matsudaira & Ishida 2021a |
| LC576798 | *Hylobates pileatus* | *cytb* | Matsudaira & Ishida 2021a |
| MN615732 | *Hylobates pileatus* | *cytb* | Leroux *et al*. 2020 |
| MN615733 | *Hylobates pileatus* | *cytb* | Leroux *et al*. 2020 |
| MN615734 | *Hylobates pileatus* | *cytb* | Leroux *et al*. 2020 |
| MN615735 | *Hylobates pileatus* | *cytb* | Leroux *et al*. 2020 |
| MN615736 | *Hylobates pileatus* | *cytb* | Leroux *et al*. 2020 |
| MN615737 | *Hylobates pileatus* | *cytb* | Leroux *et al*. 2020 |
| MN615738 | *Hylobates pileatus* | *cytb* | Leroux *et al*. 2020 |
| MN615739 | *Hylobates pileatus* | *cytb* | Leroux *et al*. 2020 |
| MN615740 | *Hylobates pileatus* | *cytb* | Leroux *et al*. 2020 |
| MN615741 | *Hylobates pileatus* | *cytb* | Leroux *et al*. 2020 |
| MN615742 | *Hylobates pileatus* | *cytb* | Leroux *et al*. 2020 |
| MN615743 | *Hylobates pileatus* | *cytb* | Leroux *et al*. 2020 |
| MN615744 | *Hylobates pileatus* | *cytb* | Leroux *et al*. 2020 |
| MN615745 | *Hylobates pileatus* | *cytb* | Leroux *et al*. 2020 |
| MN615746 | *Hylobates pileatus* | *cytb* | Leroux *et al*. 2020 |
| MN615747 | *Hylobates pileatus* | *cytb* | Leroux *et al*. 2020 |
| MN615748 | *Hylobates pileatus* | *cytb* | Leroux *et al*. 2020 |
| MN615749 | *Hylobates pileatus* | *cytb* | Leroux *et al*. 2020 |
| MN615750 | *Hylobates pileatus* | *cytb* | Leroux *et al*. 2020 |
| MN615751 | *Hylobates pileatus* | *cytb* | Leroux *et al*. 2020 |
| MN615752 | *Hylobates pileatus* | *cytb* | Leroux *et al*. 2020 |
| MN615753 | *Hylobates pileatus* | *cytb* | Leroux *et al*. 2020 |
| MN615754 | *Hylobates pileatus* | *cytb* | Leroux *et al*. 2020 |
| MN615756 | *Hylobates pileatus* | *cytb* | Leroux *et al*. 2020 |
| MN615757 | *Hylobates pileatus* | *cytb* | Leroux *et al*. 2020 |
| MN615758 | *Hylobates pileatus* | *cytb* | Leroux *et al*. 2020 |
| MN615759 | *Hylobates pileatus* | *cytb* | Leroux *et al*. 2020 |
| MN615760 | *Hylobates pileatus* | *cytb* | Leroux *et al*. 2020 |
| MN615761 | *Hylobates pileatus* | *cytb* | Leroux *et al*. 2020 |
| MN615762 | *Hylobates pileatus* | *cytb* | Leroux *et al*. 2020 |
| MN615764 | *Hylobates pileatus* | *cytb* | Leroux *et al*. 2020 |
| MN615765 | *Hylobates pileatus* | *cytb* | Leroux *et al*. 2020 |
| MN615766 | *Hylobates pileatus* | *cytb* | Leroux *et al*. 2020 |
| MN615767 | *Hylobates pileatus* | *cytb* | Leroux *et al*. 2020 |
| MN615768 | *Hylobates pileatus* | *cytb* | Leroux *et al*. 2020 |
| MN615770 | *Hylobates pileatus* | *cytb* | Leroux *et al*. 2020 |
| MN615771 | *Hylobates pileatus* | *cytb* | Leroux *et al*. 2020 |
| MN615773 | *Hylobates pileatus* | *cytb* | Leroux *et al*. 2020 |
| HQ622788 | *Hylobates klossii* | Full-length&*cytb* | Chan *et al*. 2010 |
| AJ010581 | *Hylobates klossii* | *cytb* | Unpublished |
| GU321314 | *Hylobates klossii* | *cytb* | Thinh *et al*. 2010a |
| GU321315 | *Hylobates klossii* | *cytb* | Thinh *et al*. 2010a |
| GU321316 | *Hylobates klossii* | *cytb* | Thinh *et al*. 2010a |
| GU321317 | *Hylobates klossii* | *cytb* | Thinh *et al*. 2010a |
| GU321318 | *Hylobates klossii* | *cytb* | Thinh *et al*. 2010a |
| HQ622779 | *Hylobates muelleri* | Full-length&*cytb* | Chan *et al*. 2010 |
| HQ622780 | *Hylobates muelleri* | Full-length&*cytb* | Chan *et al*. 2010 |
| HQ622781 | *Hylobates muelleri* | Full-length&*cytb* | Chan *et al*. 2010 |
| GU321308 | *Hylobates muelleri* | *cytb* | Thinh *et al*. 2010a |
| GU321309 | *Hylobates muelleri* | *cytb* | Thinh *et al*. 2010a |
| GU321310 | *Hylobates muelleri* | *cytb* | Thinh *et al*. 2010a |
| GU321311 | *Hylobates muelleri* | *cytb* | Thinh *et al*. 2010a |
| GU321312 | *Hylobates funereus* | *cytb* | Thinh *et al*. 2010a |
| GU321313 | *Hylobates abbotti* | *cytb* | Thinh *et al*. 2010a |
| LC548039 | *Hylobates abbotti* | *cytb* | Matsudaira & Ishida 2021b |
| LC548040 | *Hylobates muelleri* | *cytb* | Matsudaira & Ishida 2021b |
| LC576784 | *Hylobates abbotti* | *cytb* | Matsudaira & Ishida 2021a |
| LC576785 | *Hylobates abbotti* | *cytb* | Matsudaira & Ishida 2021a |
| LC576786 | *Hylobates abbotti* | *cytb* | Matsudaira & Ishida 2021a |
| LC576787 | *Hylobates abbotti* | *cytb* | Matsudaira & Ishida 2021a |
| LC576788 | *Hylobates abbotti* | *cytb* | Matsudaira & Ishida 2021a |
| LC576789 | *Hylobates muelleri* | *cytb* | Matsudaira & Ishida 2021a |
| LC576790 | *Hylobates muelleri* | *cytb* | Matsudaira & Ishida 2021a |
| LC576791 | *Hylobates muelleri* | *cytb* | Matsudaira & Ishida 2021a |
| LC576799 | *Hylobates sp.* | *cytb* | Matsudaira & Ishida 2021a |
| LC576800 | *Hylobates sp.* | *cytb* | Matsudaira & Ishida 2021a |
| LC576801 | *Hylobates sp.* | *cytb* | Matsudaira & Ishida 2021a |
| NC_014047 | *Symphalangus syndactylus* | Full-length&*cytb* | Matsudaira & Ishida 2010 |
| KC757411 | *Symphalangus syndactylus* | Full-length&*cytb* | Finstermeier *et al*. 2013 |
| HQ622793 | *Symphalangus syndactylus* | Full-length&*cytb* | Chan *et al*. 2010 |
| HQ622792 | *Symphalangus syndactylus* | Full-length&*cytb* | Chan *et al*. 2010 |
| HQ622798 | *Symphalangus syndactylus* | Full-length&*cytb* | Chan *et al*. 2010 |
| HQ622789 | *Symphalangus syndactylus* | Full-length&*cytb* | Chan *et al*. 2010 |
| HQ622790 | *Symphalangus syndactylus* | Full-length&*cytb* | Chan *et al*. 2010 |
| HQ622794 | *Symphalangus syndactylus* | Full-length&*cytb* | Chan *et al*. 2010 |
| HQ622796 | *Symphalangus syndactylus* | Full-length&*cytb* | Chan *et al*. 2010 |
| HQ622791 | *Symphalangus syndactylus* | Full-length&*cytb* | Chan *et al*. 2010 |
| HQ622799 | *Symphalangus syndactylus* | Full-length&*cytb* | Chan *et al*. 2010 |
| HQ622795 | *Symphalangus syndactylus* | Full-length&*cytb* | Chan *et al*. 2010 |
| HQ622797 | *Symphalangus syndactylus* | Full-length&*cytb* | Chan *et al*. 2010 |
| GU321282 | *Symphalangus syndactylus* | *cytb* | Thinh *et al*. 2010a |
| GU321283 | *Symphalangus syndactylus* | *cytb* | Thinh *et al*. 2010a |
| GU321284 | *Symphalangus syndactylus* | *cytb* | Thinh *et al*. 2010a |
| GU321285 | *Symphalangus syndactylus* | *cytb* | Thinh *et al*. 2010a |
| LC548052 | *Symphalangus syndactylus* | *cytb* | Matsudaira & Ishida 2021b |
| LC548053 | *Symphalangus syndactylus* | *cytb* | Matsudaira & Ishida 2021b |
| LC548054 | *Symphalangus syndactylus* | *cytb* | Matsudaira & Ishida 2021b |
| LC548055 | *Symphalangus syndactylus* | *cytb* | Matsudaira & Ishida 2021b |
| LC548056 | *Symphalangus syndactylus* | *cytb* | Matsudaira & Ishida 2021b |
| LC576804 | *Symphalangus syndactylus* | *cytb* | Matsudaira & Ishida 2021b |
| LC576805 | *Symphalangus syndactylus* | *cytb* | Matsudaira & Ishida 2021b |
| LC576806 | *Symphalangus syndactylus* | *cytb* | Matsudaira & Ishida 2021b |
| LC576807 | *Symphalangus syndactylus* | *cytb* | Matsudaira & Ishida 2021b |
| LC576808 | *Symphalangus syndactylus* | *cytb* | Matsudaira & Ishida 2021b |
| LC576809 | *Symphalangus syndactylus* | *cytb* | Matsudaira & Ishida 2021b |
| LC576810 | *Symphalangus syndactylus* | *cytb* | Matsudaira & Ishida 2021b |
| LC576811 | *Symphalangus syndactylus* | *cytb* | Matsudaira & Ishida 2021b |
| LC576812 | *Symphalangus syndactylus* | *cytb* | Matsudaira & Ishida 2021b |
| KF914213 | *Gorilla beringei graueri* | Full-length&*cytb* | Das *et al*. 2014 |
| NC_011120 | *Gorilla gorilla gorilla* | Full-length&*cytb* | Xu & Arnason 1996a |
| KF914214 | *Gorilla gorilla gorilla* | Full-length&*cytb* | Das *et al*. 2014 |
| KY751400 | *Homo sapiens neanderthalensis* | Full-length&*cytb* | Posth *et al*. 2017 |
| MK388903 | *Homo sapiens neanderthalensis* | Full-length&*cytb* | Mafessoni *et al*. 2020 |
| KT780370 | *Homo sapiens ssp. Denisova* | Full-length&*cytb* | Sawyer *et al*. 2015 |
| KX663333 | *Homo sapiens ssp. Denisova* | Full-length&*cytb* | Slon *et al*. 2017 |
| NC_012920 | *Homo sapiens* | Full-length&*cytb* | Andrews *et al*. 1999 |
| HM068590 | *Pan troglodytes troglodytes* | Full-length&*cytb* | Bjork *et al*. 2011 |
| KU308532 | *Pan troglodytes ellioti* | Full-length&*cytb* | Lobon *et al*. 2016 |
| GU189672 | *Pan paniscus* | Full-length&*cytb* | Zsurka *et al*. 2010 |
| NC_002083 | *Pongo abelii* | Full-length&*cytb* | Xu & Arnason 1996b |
| NC_001646 | *Pongo pygmaeus* | Full-length&*cytb* | Horai *et al*. 1992 |
| MG787545 | *Papio anubis* | Full-length&*cytb* | Roos *et al*. 2018 |
| NC_020006 | *Papio anubis* | Full-length&*cytb* | Zinner *et al*. 2013 |
| KX686496 | *Macaca mulatta vestita* | Full-length&*cytb* | Unpublished |
| AY612638 | *Macaca mulatta* | Full-length&*cytb* | Gokey *et al*. 2004 |

References of Table S2.

Andrews RM, Kubacka I, Chinnery PF, Lightowlers RN, Turnbull DM, Howell N (1999). Reanalysis and revision of the Cambridge reference sequence for human mitochondrial DNA. *Nature Genetics* 23(2):147-147.

Arnason U, Gullberg A, Xu X (1996). A complete mitochondrial DNA molecule of the white-handed gibbon, *Hylobates lar*, and comparison among individual mitochondrial genes of all hominoid genera. *Hereditas* 124(2):185-189.

Bjork A, Liu W, Wertheim JO, Hahn BH, Worobey M (2011). Evolutionary History of Chimpanzees Inferred from Complete Mitochondrial Genomes. *Molecular Biology and Evolution* 28(1):615-623.

Chan YC, Roos C, Inoue-Murayama M, *et al*. (2010). Mitochondrial Genome Sequences Effectively Reveal the Phylogeny of Hylobates Gibbons. Fleischer RC, ed. *PLoS One* 5(12):e14419.

Das R, Hergenrother SD, Soto-Calderón ID, Dew JL, Anthony NM, Jensen-Seaman MI (2014). Complete Mitochondrial Genome Sequence of the Eastern Gorilla (*Gorilla beringei*) and Implications for African Ape Biogeography. *Journal of Heredity* 105(6):846-855.

Fan PF, He K, Chen X, *et al*. (2017). Description of a new species of Hoolock gibbon (Primates: Hylobatidae) based on integrative taxonomy. *American Journal of Primatology* 9(5):e22631.

Finstermeier K, Zinner D, Brameier M, *et al.* (2013). A Mitogenomic Phylogeny of Living Primates. Stanyon R, ed. *PLoS One* 8(7):e69504.

Gokey NG, Cao Z, Pak JW, *et al.* (2004). Molecular analyses of mtDNA deletion mutations in microdissected skeletal muscle fibers from aged rhesus monkeys. *Aging Cell* 3(5):319-326.

Horai S, Satta Y, Hayasaka K, *et al.* (1992). Man’s place in hominoidea revealed by mitochondrial DNA genealogy. *Journal of Molecular Evolution* 35(1):32-43.

Leroux N, Nouhin J, Prak S, *et al.* (2020). Prevalence and Phylogenetic Analysis of Hepatitis B in Captive and Wild-Living Pileated Gibbons (Hylobates pileatus) in Cambodia. *International Journal of Primatology* 41(4):634-653.

Lobon I, Tucci S, De Manuel M, *et al.* (2016). Demographic History of the Genus Pan Inferred from Whole Mitochondrial Genome Reconstructions. *Genome Biology and Evolution* 8(6):2020-2030.

Mafessoni F, Grote S, de Filippo C, *et al.* (2020). A High-Coverage Neandertal Genome from Chagyrskaya Cave. *Proceedings of the National Academy of Sciences of the United States of America* 117(26):15132–15136

Matsudaira K, Ishida T (2010). Phylogenetic relationships and divergence dates of the whole mitochondrial genome sequences among three gibbon genera. *Molecular Phylogenetics and Evolution* 55(2):454-459.

Matsudaira K, Maeda Y, Shidehara N, *et al.* (2021a). Species identification of captive gibbons in Japan by using mitochondrial DNA (cytochrome b) sequences. *Japanese Journal of Zoo and Wildlife Medicine* 26(2):35-42.

Matsudaira K, Ishida T (2021). Divergence and introgression in small apes, the genus Hylobates, revealed by reduced representation sequencing. *Heredity* 127(3):312-322.

Nie WH, Wang JH, Su WT, *et al.* (2018). Species identification of crested gibbons (Nomascus) in captivity in China using karyotyping- and PCR-based approaches. *Zoological Research* 39(5): 356–63.

Posth C, Wißing C, Kitagawa K, *et al.* (2017). Deeply divergent archaic mitochondrial genome provides lower time boundary for African gene flow into Neanderthals. *Nature Communications* 8(1):16046.

Roos C, Chuma IS, Collins DA, *et al.* (2018). Complete Mitochondrial Genome of an Olive Baboon (Papio Anubis) from Gombe National Park, Tanzania. *Mitochondrial Dna B* 3(1):177-178.

Roos C, Portela Miguez R, Zinner D, *et al.* (2022). Importance of genetic data from type specimens: The questionable type locality of southern white-cheeked gibbon, Nomascus siki (Delacour, 1951). *Zoological Research* 43(4):666-670.

Sawyer S, Renaud G, Viola B, *et al.* (2015). Nuclear and Mitochondrial DNA Sequences from Two Denisovan Individuals. *PNAS* 112(51):15696-15700.

Slon V, Viola B, Renaud G, *et al.* (2017). A Fourth Denisovan Individual. *Science Advances* 3:e1700186.

Thinh VN, Mootnick AR, Geissmann T, *et al.* (2010a). Mitochondrial evidence for multiple radiations in the evolutionary history of small apes. *BMC Evolutionary Biology* 10(1):74.

Thinh VN, Rawson B, Hallam C, *et al.* (2010b). Phylogeny and distribution of crested gibbons (genus Nomascus) based on mitochondrial cytochrome b gene sequence data. *American Journal of Primatology* 72(12):1047-1054.

Xu XF, Arnason U (1996a). A complete sequence of the mitochondrial genome of the western lowland gorilla. *Molecular Biology and Evolution* 13(5):691-698.

Xu XF, Arnason U (1996b). The Mitochondrial DNA Molecule of Sumatran Orangutan and a Molecular Proposal for Two (Bornean and Sumatran) Species of Orangutan. *Journal of Molecular Evolution* 43(5):431-437

Zinner D, Wertheimer J, Liedigk R, *et al.* (2013). Baboon phylogeny as inferred from complete mitochondrial genomes. *American Journal of Physical Anthropology* 150(1):133-140.

**Table S3** Comparison of estimated divergence times for different partitions

| Divergence | full-length | | CDS | | *cytb* | | 1st-2rd codon | | 3rd codon | | rRNAs | |
| --- | --- | --- | --- | --- | --- | --- | --- | --- | --- | --- | --- | --- |
|  | Mean | 95%HPD | Mean | 95%HPD | Mean | 95%HPD | Mean | 95%HPD | Mean | 95%HPD | Mean | 95%HPD |
| *Macaca&Pango*-Hominoidea† | 37.02 | 30.09-43.69 | 37.03 | 30.31-43.83 | 31.71 | 24.50-39.04 | 35.49 | 28.63-42.25 | 36.56 | 29.84-43.69 | 34.82 | 27.61-41.89 |
| Hominidae-Hylobatidae | 20.12 | 16.50-23.95 | 19.48 | 16.04-23.29 | 18.25 | 13.86-22.45 | 18.27 | 14.69-21.72 | 21.53 | 17.59-25.71 | 19.78 | 15.60-23.93 |
| Hylobatidae | 7.22 | 5.90-8.59 | 7.00 | 5.72-8.35 | 7.33 | 5.59-9.32 | 6.73 | 5.33-8.11 | 7.07 | 5.63-8.42 | 7.38 | 5.71-9.12 |
| *Hylobates-Symphalangus&Hoolock* | 6.22 | 5.08-7.41 | 6.19 | 5.04-7.37 | 6.28 | 4.78-8.14 | 6.14 | 4.88-7.44 | 6.12 | 4.95-7.35 | 6.69 | 5.21-8.30 |
| *Symphalangus-Hoolock* | 5.33 | 4.34-6.37 | 5.46 | 4.43-6.54 | 5.90 | 4.30-7.53 | 5.59 | 4.34-6.76 | 5.37 | 4.25-6.45 | 6.16 | 4.75-7.66 |
| *Nomascus* | 2.14 | 1.72-2.57 | 2.10 | 1.66-2.53 | 3.10 | 2.23-3.9 | 2.26 | 1.63-2.89 | 1.90 | 1.50-2.34 | 2.41 | 1.67-3.20 |
| *N. hainanus-N. nasutus*‡ | / | / | / | / | 2.36 | 1.62-3.14 | / | / | / | / | / | / |
| *N. concolor-N.spp.* | 1.68 | 1.36-2.01 | 1.67 | 1.33-2.00 | 2.03 | 1.44-2.67 | 2.26 | 1.63-2.89 | 1.52 | 1.21-1.8 | 1.85 | 1.30-2.42 |
| *N. gabriellae (N.annamensis)- N. leucogenys&N. siki*‡ | 1.14 | 0.92-1.38 | 1.10 | 0.88-1.34 | 1.23 | 0.85-1.64 | 1.24 | 0.91-1.58 | 0.98 | 0.77-1.20 | 1.20 | 0.80-1.63 |
| *N. gabriellae-N.annamensis* | / | / | / | / | 0.45 | 0.27-0.65 | / | / | / | / | / | / |
| *N. leucogenys-N. siki* | 0.38 | 0.30-0.47 | 0.34 | 0.26-0.42 | 0.64 | 0.40-0.90 | 0.49 | 0.34-0.64 | 0.28 | 0.21-0.35 | 0.34 | 0.17-0.51 |
| *N. concolor* | 0.20 | 0.15-0.25 | 0.16 | 0.11-0.20 | 0.26 | 0.15-0.39 | 0.23 | 0.13-0.35 | 0.14 | 0.09-0.18 | 0.27 | 0.12-0.44 |

The estimated divergence time was millions years ago.

†Node used for calibration

‡Only *cytb* dataset includes *N. nasutus* and *N.annamensis*

**Table S4** Fossil sites of gibbon family used in our study.

| ID | Species | Latitude (N) | Longitude (E) | Site | Province | Geological time | Reference |
| --- | --- | --- | --- | --- | --- | --- | --- |
| 1 | *Junzi imperialis* | 34.12 | 108.93 | Shenheyuan | Xian city, Shaanxi | Holocene | Turvey *et al*. 2018 |
| 2 | *Nomascus sp.* | 22.61 | 107.32 | Longlinshan Cave | Chongzuo, Guangxi | Holocene | unpublished data |
| 3 | *Nomascus sp.* | 29.03 | 106.66 | Yemao Cave | Qijiang district, Chongqing | Holocene | unpublished data |
| 4 | *Hylobates sp.* | 26.71 | 117.50 | Yanzai Cave | Jiangle county, Fujian | Late Pleistocene | You *et al*. 1996 |
| 5 | *Hylobates sp.* | 26.79 | 119.92 | Huangguashan | Xiapu county, Fujian | Holocene | Yang 2019 |
| 6 | *Hylobates sp.* | 27.79 | 113.82 | Yangjiawan 1 | Shangli county, Jiangxi | Late Pleistocene | Zou *et al*. 2016 |
| 7 | *Hylobates sp.* | 33.13 | 110.22 | Huanglong Cave | Xunxi county, Hubei | Middle to Late Pleistocene | Wu *et al*. 2007 |
| 8 | *Hylobates sp.* | 30.66 | 110.07 | Yangjiapo Cave | Jianshi county, Hubei | Late Pleistocene | Lu 2010 |
| 9 | *Hylobates cf. concolor* | 25.65 | 111.49 | Tangbeicun Cave | Dao county, Hunan | Late Pleistocene | Chen 1986 |
| 10 | *Hylobates sp.* | 28.80 | 109.67 | Dongpaoshan | Baojing county, Hunan | Early Pleistocene | Wang *et al*. 1982 |
| 11 | *Nomascus concolor* | 22.71 | 111.83 | Xiashan Cave | Luoding city, Guangdong | Middle Pleistocene | Song *et al*. 1989 |
| 12 | *Hylobates sp.* | 24.07 | 114.99 | Bishou Cave | Dongyuan county, Guangdong | Late Pleistocene | Peng *et al*. 2011 |
| 13 | *Hylobates sp.* | 24.15 | 112.93 | Litangshan | Yingde city, Guangdong | Middle Pleistocene | Zhang *et al*. 1998 |
| 14 | *Hylobatidae* | 23.59 | 107.01 | Zhongshan | Baise city, Guangxi | Holocene | Chen *et al*. 2017 |
| 15 | *Nomascus concolor* | 23.48 | 108.57 | Nongrong Cave | Shanglin county, Guangxi | Late Pleistocene | Gu 1986 |
| 16 | *Hylobates sp.* | 25.29 | 110.30 | Baojiyan | Guilin city, Guangxi | Late Pleistocene | Gu 1986 |
| 17 | *Nomascus concolor* | 25.31 | 110.24 | Taipingyan | Guilin city, Guangxi | Late Pleistocene | Gu 1986 |
| 18 | *Hylobates sp.* | 24.78 | 110.50 | / | Yangshuo county, Guangxi | Late Pleistocene | Gu 1986 |
| 19 | *Hoolock sp.* | 24.50 | 110.39 | / | Lipu county, Guangxi | Late Pleistocene | Gu 1986 |
| 20 | *Hylobates sp.* | 24.34 | 109.45 | / | Liuzhou city, Guangxi | Late Pleistocene | Gu 1986 |
| 21 | *Nomascus concolor* | 24.25 | 109.31 | Shaoniyan Cave | Liujiang county, Guangxi | Late Pleistocene | Gu 1986 |
| 22 | *Nomascus concolor* | 24.25 | 109.27 | Zhongmenyan Cave | Liujiang county, Guangxi | Late Pleistocene | Gu 1986 |
| 23 | *Nomascus concolor* | 24.17 | 109.31 | Baishanyan Cave | Liujiang county, Guangxi | Late Pleistocene | Gu 1986 |
| 24 | *Hoolock sp.* | 24.17 | 109.71 | / | Liujiang county, Guangxi | Late Pleistocene | Gu 1986 |
| 25 | *Nomascus concolor* | 24.46 | 109.13 | Lingyan Cave | Liujiang county, Guangxi | Late Pleistocene | Gu 1986 |
| 26 | *Nomascus sp.* | 22.29 | 107.51 | Juyuan Cave | Chongzuo, Guangxi | Early Pleistocene | Wang *et al*. 2015 |
| 27 | *Hylobates sp.* | 22.28 | 107.51 | Sanhe Cave | Chongzuo, Guangxi | Early Pleistocene | Zhang *et al*. 2018 |
| 28 | *Hylobates sp.* | 22.29 | 107.51 | Zhiren Cave | Chongzuo, Guangxi | Late Pleistocene | Zhang *et al*. 2018 |
| 29 | *Nomascus sp.* | 22.30 | 107.52 | Shuangtan Cave | Chongzuo, Guangxi | Middle Pleistocene | Zhang *et al*. 2018 |
| 30 | *Nomascus sp.* | 22.38 | 107.37 | Yixiantian | Chongzuo, Guangxi | Middle Pleistocene | Zhang *et al*. 2018 |
| 31 | *Nomascus sp.* | 22.38 | 107.37 | Yugong | Chongzuo, Guangxi | Late Pleistocene | Zhang *et al*. 2018 |
| 32 | *Nomascus sp.* | 22.38 | 107.37 | Baxian | Chongzuo, Guangxi | Late Pleistocene | Zhang *et al*. 2018 |
| 33 | *Hylobates sp.* | 22.57 | 107.33 | Yanli Cave | Chongzuo, Guangxi | Late Pleistocene | Yao 2019 |
| 34 | *Nomascus sp.* | 22.64 | 107.62 | Baikong Cave | Chongzuo, Guangxi | Early Pleistocene | Zhang *et al*. 2018 |
| 35 | *Hylobates sp.* | 22.69 | 107.24 | Black Cave | Chongzuo, Guangxi | Middle Pleistocene | Zhang *et al*. 2018 |
| 36 | *Nomascus concolor* | 24.88 | 105.13 | Xiangbo township | Longlin county, Guangxi | Late Pleistocene | Gu 1986 |
| 37 | *Hylobates sp.* | 24.70 | 108.35 | Feishuyan | Yizhou city, Guangxi | Late Pleistocene | Gu 1986 |
| 38 | *Hylobates sp.* | 24.30 | 107.11 | Nongmeshan | Bama county, Guangxi | Middle Pleistocene | Chang *et al*. 1975 |
| 39 | *Nomascus concolor* | 23.98 | 108.00 | Jiulengshan | Du'an county, Guangxi | Late Pleistocene | Zhao 1981 |
| 40 | *Hylobates sp.* | 23.58 | 107.00 | Mehui Cave | Baise city, Guangxi | Early Pleistocene | Wang *et al*. 2007 |
| 41 | *Nomascus hainanus* | 18.28 | 109.52 | Luobi Cave | Sanya city, Hainan | Late Pleistocene to Holocene | Hao &Wang 1992 |
| 42 | *Hylobatidae* | 29.44 | 108.79 | Laowuji | Qianjiang district, Chongqing | Middle to Late Pleistocene | Website |
| 43 | *Hylobatidae* | 30.85 | 109.64 | Yumi Cave | Wushan county, Chongqing | Middle Pleistocene to Holocene | Wei *et al*. 2015 |
| 44 | *Hylobates cf. sericus* | 30.60 | 109.14 | Xinglong Cave | Fengjie county, Chongqing | Middle Pleistocene | Gao *et al*. 2003 |
| 45 | *Hylobates sp.* | 28.38 | 108.86 | Dongwan | Xiushan county, Chongqing | Early to Middle Pleistocene | Chen *et al*. 2012 |
| 46 | *Nomascus concolor* | 28.03 | 106.05 | Yemao Cave | Gulin county, Sichuan | Holocene | Yang & Yang 1995 |
| 47 | *Hylobates sp.* | 28.21 | 106.74 | Yanhui Cave | Tongzi county, Guizhou | Middle Pleistocene | Wu & Wang 1975 |
| 48 | *Hylobates sp.* | 27.72 | 105.37 | Mawokou Cave | Bijie city, Guizhou | Late Pleistocene | Zhao *et al*. 2016 |
| 49 | *Hylobates sp.* | 24.25 | 102.22 | Laolong Cave | Eshan district, Yunnan | Late Pleistocene | Bai 1998 |
| 50 | *Hylobates sp.* | 25.92 | 101.77 | Baozidongjing | Yuanmou county, Yunnan | Pliocene | Yunnan Provincial Museum *et al*. 1989 |
| 51 | *Nomascus concolor* | 23.42 | 104.68 | Xianren Cave | Xichou county, Yunnan | Late Pleistocene | Chen *et al*. 1978 |

| Note: As a result of taxonomic changes, the *Hylobates sp.* recorded in previous papers likely represents the family Hylobatidae. |
| --- |
| References of Table S4. |
| Bai ZL (1998). A prelminary study on the prehistoric site of Laolongdong. *Acta Anthropologica Sinica* 17(3): 212-229. |
| Chen DZ, Qi GQ (1978). Human fossils from Xichou, Yunnan province, and the co-occurring mammal fauna. *Vertebrate Palasiatica* 16(1): 33-46. |
| Chen J, Wang W, LiW, *et al.* (2017). Research into faunal remains excavated from the rock shelter area of the Zhongshan site, Tiandong county, Guangxi, South China. *Acta Anthropologica Sinica* 36(4): 527-536. |
| Chen SH, He CD, Qin L, *et al.* (2012). New Pleistocene mammalian fauna from Xiushan county, Chongqing. In: Dong W ed. Proceedings of the Thirteenth Annual Meeting of the Chinese Society of Vertebrate Paleontology; Aug 2012, Inner Mongolia Autonomous Region, China. China Ocean Press, Beijing. |
| Chen XB (1986). New material of Pleistocene mammalian fossil found in Hunan Province. *Vertebrate Palasiatica* 24(3): 242-244. |
| Gao X, Huang WB, Xu ZQ, *et al.* (2003). Ancient human fossils and ivory carvings from 120,000-150,000 years ago excavated from Xinglong Cave, Three Gorges. *Science Bulletin* 48(23): 2466-2472. |
| Gu YM (1986). Preliminary research on the fossil gibbon of Pleistocene China. *Acta Anthropologica Sinica* 5(3): 208-219. |
| Hao SD, Wang DX, Sun JP, *et al.* (1994). Report on the excavation of the Sanya hominld site in 1992. *Acta Anthropologica Sinica* 13(2): 117-125. |
| Lu CQ (2010). Late Pleistocene mammalian fauna from Yangjiapo Cave, Jianshi, Hubei.In: Dong W ed. Proceedings of the Twelfth Annual Meeting of the Chinese Society of Vertebrate Paleontology; 13 Sep 2010, Shangdong, China. China Ocean Press, Beijing. |
| Peng PX, Li BX, Bian G*G, et al.* (2011). Second study about mammal fossils of the Bishou Cave in Dongjiang River Valleys, southern Nanling Mountains, China. *Acta Geologica Sinica* 85(12):2031-2038. |
| Song FY, Zhang ZH, Guo XF, *et al.* (1989). A preliminary report on the excavation of cave site at Fanzengshanyan and Xiashandong of Luoding, Guangdong. *Acta Anthropological Sinica* 8(2): 118-122. |
| Turvey ST, Bruun K, Ortiz A, *et al.* (2018). New genus of extinct Holocene gibbon associated with humans in Imperial China. *Science* 360. 1346-1349. |
| Wang LH, Lin YF, Chang SW, *et al.* (1982). Mammalian fossils found in northwest part of Hunan province and their significance. *Vertebrate Palasiatica* 20(4): 350-358. |
| Wang W, Tian F, Mo JY. (2007). Recovery of Gigantopithecus blacki fossils from the Mohui cave in the Buding basin, Guangxi, South China. *Acta Anthropologica Sinica* 26(4):329-343. |
| Wang Y, Jin CZ, Pan WS, *et al.* (2015). The Early Pleistocene Gigantopithecus-Sinomastodon fauna from Juyuan karst cave in Boyue Mountain, Guangxi, South China. *Quaternary International* 434:4–16. |
| Wei GB, Huang WB, Boeda E, *et al.* (2015). Recent discovery of a unique paleolithic industry from the yumidong cave site in the three gorges region of yangtze river, southwest China. *Quaternary International* 434(PT.A), 107-120. |
| Wu ML, Wang LH, Zhang YY, *et al.* (1975). Ancient human fossils and their cultural remains found in Tongzi, Guizhou. *Vertebrate Palasiatica* 13(1): 14-23. |
| Wu XZ, Wu XJ, Chen MH, *et al.* 2007. The 2006 excavation of Huanglong Cave in Yunxi county, Hubei. *Acta Anthropologica Sinica* 2007, 26(3): 193-205. |
| Yang S (2019). Stable Isotope Analysis on animal skeletal remains at Huangguashan Site. master's thesis. Xiamen: Xiamen Universiy. |
| Yang XL, Yang DH, Zhao GL, *et al.* (1995). Researches of Ailuropoda-Stegodon Fauna from Gulin, China. Chongqing Publish Group, Chongqing. |
| Yao YY, Liao W, Bae CJ, *et al.* (2020). *New discovery of Late Pleistocene modern human teeth in Chongzuo, Guangxi, southern China.* *Quaternary International* 563:5-12. |
| You YZ, Cai BQ (1996). Stratigraphic division, fossil mammals and environment of the Pleistocene in Fujian province. *Acta Anthropologica Sinica* 15(4): 335-346. |
| Yunnan Provincial Museum, Yuanmou People's Exhibition Hall, *et al.* (1989). Briefing on the excavation of fossilised ancient hominids from Zhupeng, Yuanmou, Yunnan. *Prehistoric Research* 00: 46-50. |
| Zhang YQ, Jin CZ, Wang Y, *et al.* (2018). Fossil gibbons (Mammalia, Hylobatidae) from the Pleistocene of Chongzuo, Guangxi, China. *Vertebrata Palasiatica* 56(3): 248-263. |
| Zhang Y Y, Wang L H, Dong X R, *et al.* (1975). Discovery of a Gigantopithecus tooth from Bama district in Kwangsi. *Vertebrate Palasiatica* 13(3): 148-153. |
| Zhang ZH, Jin ZW, Qu JG, *et al.* (1998). Discovery of the fauna from Litang Mountain, Jiulong, Yingde, Guangdong. *Jianghan Archaeology* (1): 28-29. |
| Zhao LX, Zhang LZ, Du BP, *et al.* (2016). New discovery of Hunan fossils and associated mammal fauna from Mawokou Cave in Bijie, Guizhou province of southern China. *Acta Anthropologica Sinica* 35(1): 24-35. |
| Zhao ZR, Liu XS, Wang LH (1981). Human fossil and associated fauna of Jiulengshan hill, Guangxi. *Vertebrate Palasiatica* 1981, 19(1): 45-54. |
| Zou SL, Chen X, Zhang B, *et al.* (2016). Preliminary report on the Late Pleistocene mammalian fauna from Shangli County, Pingxiang, Jiangxi Province. *Acta Anthropologica Sinica* 35(1): 109-120. |

**Table S5** Diversity indices calculated using mitochondrial genomes for different species in gibbon family.

| Species | n | H | S | π | H_d_ | k | θ_S_ | θ_π_ | N_ef_ (θ_S_) | N_ef_(θ_π_) |
| --- | --- | --- | --- | --- | --- | --- | --- | --- | --- | --- |
| *Hylobates muelleri* | 3 | 3 | 402 | 0.01671±0.00193 | 1.000±0.272 | 268.000 | 56.00±33.82 | 56.00±42.18 | 8539 | 8539 |
| *Hylobates agilis* | 5 | 5 | 394 | 0.01310±0.00239 | 1.000±0.016 | 199.000 | 53.76±27.08 | 56.40±34.56 | 8197 | 8600 |
| *Symphalangus syndactylus* | 13 | 12 | 331 | 0.00618±0.00148 | 0.987±0.035 | 89.282 | 31.26±12.05 | 25.59±13.51 | 4766 | 3902 |
| *Hylobates moloch* | 4 | 4 | 132 | 0.00518±0.00142 | 1.000±0.177 | 83.333 | 20.18±11.19 | 23.83±15.97 | 3077 | 3634 |
| *Hoolock leuconedys* | 12 | 12 | 168 | 0.00466±0.00054 | 1.000±0.034 | 53.182 | 21.52±8.58 | 21.82±11.66 | 3282 | 3327 |
| *Nomascus gabriellae* | 2 | 2 | 70 | 0.00432±0.00216 | 1.000±0.250 | 70.000 | 19.00±13.78 | 19.00±19.49 | 2897 | 2897 |
| *Hylobates lar* | 16 | 16 | 261 | 0.00337±0.00103 | 1.000±0.022 | 49.458 | 18.99±7.09 | 12.18±6.51 | 2895 | 1856 |
| *Nomascus leucogenys* | 5 | 5 | 111 | 0.00319±0.00084 | 1.000±0.016 | 49.200 | 13.44±7.03 | 12.40±7.91 | 2049 | 1891 |
| *Nomascus siki* | 5 | 5 | 85 | 0.00285±0.00047 | 1.000±0.126 | 40.600 | 11.04±5.84 | 11.20±7.18 | 1683 | 1708 |
| *Nomascus concolor* | 9 | 8 | 91 | 0.00253±0.00031 | 0.972±0.064 | 33.482 | 13.61±5.99 | 13.89±7.82 | 2076 | 2118 |
| *Hoolock hoolock* | 10 | 9 | 140 | 0.00241±0.00075 | 0.978±0.054 | 39.489 | 11.67±5.04 | 9.09±5.17 | 1779 | 1386 |
| *Hoolock tianxing* | 3 | 3 | 22 | 0.00098±0.00035 | 1.000±0.272 | 14.667 | 2.00±1.51 | 2.00±1.89 | 305 | 305 |
| *Hylobates pileatus* | 3 | 3 | 17 | 0.00071±0.00024 | 1.000±0.272 | 11.333 | 0.67±0.67 | 0.67±0.83 | 102 | 102 |

**Table S6** Comparison of D-loop genetic diversity with other primates

| Species | n | Length | H | S | π | H_d_ | References |
| --- | --- | --- | --- | --- | --- | --- | --- |
| *Nomascus concolor* | 37 | 1025 | 6 | 16 | 0.00501 | 0.384 | This study |
| *Nomascus hainanus* | 6 | 202 | 4 | 5 | 0.00820 | 0.600 | Li *et al*. 2010 |
|  | 12 | 1005 | 2 | 5 | 0.00271 | 0.545 | Han 2019 |
| *Nycticebus bengalensis* | 59 | 386 | 36 | 46 | 0.01538 | 0.975 | He 2018 |
| *Nycticebus pygmaeus* | 15 | 400 | / | / | 0.00516 | / | Zhu *et al*. 2015 |
| *Rhinopithecus bieti* | 157 | 401 | 30 | / | 0.03400 | 0.944 | Liu *et al*. 2007 |
| *Rhinopithecus brelichi* | 288 | 866 | 11 | 32 | 0.00413 | 0.517 | Wang 2022 |
| *Macaca thibetana* | 14 | 477 | / | 25 | 0.01385 | 0.484 | Zhong *et al*. 2013 |
|  | 16 | 477 | / | 38 | 0.02911 | 0.783 | Zhong *et al*. 2013 |
| *Trachypithecus leucocephalus* | 9 | 396 | 7 | 16 | 0.01167 | 0.944 | Cao 2005 |

| References of Table S6. |
| --- |
| Li ZG, Wei FW, Zhou J (2010). Mitochondrial D-loop region analysis and population restoration of the *N. hainanus*. *Biodiversity Science* 18(5):523-527. |
| Han L (2019). Study on Population Genetic Diversity of Hainan gibbon (*Nomascus hainanus*). master's thesis. Guiyang (Guizhou): Normal University. |
| He X (2018). Genetic differentiation in captive populations of Nycticebus bengalensis based on mitochondrial DNA. master's thesis. Ya'an (Sichuan): Agricultural University. |
| Zhu WL, Zhang D, Gao WR, Wang ZK (2014). Genetic analysis of the D-loop and *cytb* gene in the genus *Nycticebus*. *JOURNAL OF BIOLOGY* 30(05):1-5. |
| Liu ZJ, Ren BP, Wei FW, Long YC, Hao YL, Li M (2007). Phylogeography and population structure of the Yunnan snub-nosed monkey (Rhinopithecus bieti) inferred from mitochondrial control region DNA sequence analysis. *Molecular Ecology* 16(16):3334-3349. |
| Wang S (2022). Population Genetics Study of Rhinopithecus brelichi. master's thesis. Guiyang (Guizhou): Normal University. |
| Zhong LJ, Zhang MW, Yao YF, *et al.* (2013).Genetic diversity of two Tibetan macaque (Macaca thibetana) populations from Guizhou and Yunnan in China based on mitochondrial DNA D-loop sequences. *Genes & Genomics* 35(2):205-214. |
| Cao HM (2005). The Study on Mitochondrial DNA Genetic Diversity of the White-headed Leaf monkey (Trachypithecus leucocephalus). master's thesis. Guilin (Guangxi): Agricultural University. |

**Table S7** Diversity indices for different sites calculated by *cytb*.

| Indice | Lincang, Yunnan, China | Simao, Yunnan, China | Wayao, Yunnan, China | Bokeo, Laos | Vietnam |
| --- | --- | --- | --- | --- | --- |
| n | 2 | 3 | 2 | 2 | 7 |
| H | 1 | 3 | 2 | 2 | 7 |
| S | 0 | 11 | 3 | 5 | 11 |
| π | / | 0.00661 | 0.00263 | 0.00439 | 0.00326 |
| H_d_ | / | 1 | 1 | 1 | 1 |
| k | / | 7.333 | 3 | 5 | 3.714 |

**Table S8** Selection pressures on *N. concolor* and *N. hainanus*

| Test | lnL | ω（dN/dS） | np | 2Δl | p-value |
| --- | --- | --- | --- | --- | --- |
| M0 | -20553.76653 | 0.08105 | 43 | / | / |
| Test 1 *N. hainanus* as foreground | -20553.76894 | ω_0_=0.08105 | 45 | -0.004812 | / |
|  |  | ω1=30.17657 |  |  |  |
| Test 2 *N. concolor* as foreground | -20553.53448 | ω_0_=0.07806 | 45 | 0.464098 | 0.792906477 |
|  |  | ω1=0.08858 |  |  |  |

**Table S9** Selection pressures on different branches of *N. concolor*

| Test | lnL | ω（dN/dS） | np | 2Δl | p-value |
| --- | --- | --- | --- | --- | --- |
| M0 | -15213.23861 | ω_0_=0.09754 | 17 | / | / |
| Test 1 Subspecies classification | -15212.06872 | ω_0_=0.09307 | 20 | 2.33979 | 0.50494 |
|  |  | ω_1_=0.25676 |  |  |  |
|  |  | ω_2_=0.12071 |  |  |  |
|  |  | ω_3_=0.00010 |  |  |  |
| Test 2 Geographic locations | -15212.32452 | ω_0_=0.10213 | 20 | 1.82816 | 0.60882 |
|  |  | ω_1_=0.12736 |  |  |  |
|  |  | ω_2_=0.00010 |  |  |  |
|  |  | ω_3_=0.00010 |  |  |  |
| Test 3 Phylogenetic branches | -15212.71683 | ω_0_=0.12957 | 19 | 1.04355 | 0.59348 |
|  |  | ω_1_=0.06419 |  |  |  |
|  |  | ω_2_=0.10639 |  |  |  |

Note: Regarding the setup of the foreground branch, (1) Test 1 is set according to the classification of the subspecies, with *N. c. furvogaster* labelled as #1, *N. c. jingdongensis* as #2, and *N. c. concolor* as #3. (2) Test 2 was set according to the sampling location, with Simao labelled as #1, Honghe labelled as #2, and Lincang labelled as #3. (3) Test3 was set according to the location in the phylogenetic tree, and the clade labelled in $ 1 included *N. c. furvogaster 2* and *N. c. furvogaster 1*, $2 included *Nomascus concolor* HQ622808, *N. c. furvogaster 4* and *N. c. concolor 1*. Remaining branches were background branches.
